# High throughput single cell long-read sequencing analyses of same-cell genotypes and phenotypes in human tumors

**DOI:** 10.1038/s41467-023-39813-7

**Published:** 2023-07-11

**Authors:** Cheng-Kai Shiau, Lina Lu, Rachel Kieser, Kazutaka Fukumura, Timothy Pan, Hsiao-Yun Lin, Jie Yang, Eric L. Tong, GaHyun Lee, Yuanqing Yan, Jason T. Huse, Ruli Gao

**Affiliations:** 1 Department of Biochemistry and Molecular Genetics, Northwestern University Feinberg School of Medicine, Chicago, IL 60611 USA; 2 Center for Cancer Genomics, Robert H. Lurie Cancer Center, Northwestern University, Chicago, IL 60611 USA; 3 Center for RNA Therapeutics, Houston Methodist Research Institute, Houston, TX 77030 USA; 4 Department of Translational Molecular Pathology, University of Texas MD Anderson Cancer Center, Houston, TX 77030 USA; 5 The Driskill Graduate Program, Northwestern University, Chicago, IL 60611 USA; 6 Department of Radiation Oncology, New York University Langone School of Medicine, New York, NY 100167 USA; 7 School of Engineering, University of Texas at Austin, Austin, TX 78712 USA; 8 Center for Genetic Medicine, Northwestern University Feinberg School of Medicine, Chicago, IL 60611 USA; 9 Department of Surgery, Northwestern University Feinberg School of Medicine, Chicago, IL 60611 USA; 10 Department of Pathology and Translational Molecular Pathology, University of Texas MD Anderson Cancer Center, Houston, TX 77030 USA

**Keywords:** Genome assembly algorithms, RNA sequencing, Transcriptomics, RNA splicing, Software

## Abstract

Single-cell nanopore sequencing of full-length mRNAs transforms single-cell multi-omics studies. However, challenges include high sequencing errors and dependence on short-reads and/or barcode whitelists. To address these, we develop scNanoGPS to calculate same-cell genotypes (mutations) and phenotypes (gene/isoform expressions) without short-read nor whitelist guidance. We apply scNanoGPS onto 23,587 long-read transcriptomes from 4 tumors and 2 cell-lines. Standalone, scNanoGPS deconvolutes error-prone long-reads into single-cells and single-molecules, and simultaneously accesses both phenotypes and genotypes of individual cells. Our analyses reveal that tumor and stroma/immune cells express distinct combination of isoforms (DCIs). In a kidney tumor, we identify 924 DCI genes involved in cell-type-specific functions such as *PDE10A* in tumor cells and *CCL3* in lymphocytes. Transcriptome-wide mutation analyses identify many cell-type-specific mutations including *VEGFA* mutations in tumor cells and *HLA-A* mutations in immune cells, highlighting the critical roles of different mutant populations in tumors. Together, scNanoGPS facilitates applications of single-cell long-read sequencing technologies.

## Abstract (web summary)

There is a need for methods that allow the analysis of single-cell long-read sequencing data without depending on known barcode lists or short-read sequencing. Here, the authors develop scNanoGPS, a tool that can independently deconvolute long reads into single cells and single molecules, and apply it on tumour and cell line data.

## Introduction

Human tumors represent complex ecological systems of diverse cell types with dynamic genetic evolution and phenotypic remodeling^1–3^. However, there is a lack of robust methods for tracking both genotypes (e.g., mutations) and phenotypes (e.g., gene expressions, isoforms) of individual cells to precisely trace the cellular and molecular dynamics during tumor evolution and treatment response. Long-read single-cell sequencing of full-length RNAs is transforming single-cell multi-omics analysis through direct measurement of nucleotide sequences of whole gene bodies without algorithmic reconstructions^4–7^. Nowadays, high throughput long-read single-cell sequencing methods take advantage of droplet barcoding systems (commonly, 10× Genomics Chromium system) to barcode full-length cDNAs of single cells and sequence them on ultra-high yield third-generation sequencing (TGS) platforms^5–8^. Of note, the Oxford Nanopore Technology (ONT) platform, PromethION, can yield ~100 million reads per flow cell, providing adequate coverage of thousands of single-cell transcriptomes. The PacBio system Sequel II system can yield ~8 million high-fidelity reads, which can measure hundreds of single-cell transcriptomes. However, the broad applications of this powerful technology are computationally challenged due to the complexity of calculating same-cell multi-omics from these long-read data. Moreover, due to higher error rates in cell barcodes (CBs) and unique molecule identifiers (UMIs) compared to next-generation sequencing (NGS), previous methods rely on generating paralleled NGS short-read data to guide the deconvolution of CBs and UMIs^5,8^, which can drastically increase experimental costs and computational complexity and often results in partial usage of data. Recently, two methods called Sockeye (https://github.com/nanoporetech/Sockeye) and BLAZE^9^ were released to detect CBs without the usage of paralleled NGS data. However, both methods relied on a theoretical barcode whitelist (10× Genomics states there are ~3.6 million unique sequences for 3′ GEX with v3 chemistry). The whitelist dependence makes both methods suboptimal because the manufactured barcodes may deviate from theoretical random combinations, particularly, the pool size reaches millions of molecules that are 2 edit-distance apart. Further, different versions of protocols may have different whitelists, and misusages of wrong whitelists are not easy to tell due to their similarities. Therefore, a robust computational tool for analyzing high throughput single-cell long-read data is still missing.

In this study, we developed a computational tool, scNanoGPS (**s**ingle **c**ell **Nano**pore sequencing analysis of **G**enotypes and **P**henotypes **S**imultaneously), to perform completely independent deconvolution of error-prone long-reads into single-cells and single-molecules and calculate both genotypes and phenotypes in individual cells from high throughput single cell nanopore RNA sequencing (scNanoRNAseq) data. In concert with this tool, we removed the NGS sequencing steps and increased throughput to 3000–6000 transcriptomes per flow cell (PromethION). We demonstrated the accuracy and robustness of scNanoGPS and identified cell-type-specific isoforms and mutations in addition to gene expression profiles, enabling synchronous cell-lineage (genotype) and cell-fate (phenotype) tracing in human tissues.

## Results

### Computational workflow of scNanoGPS in analyzing scNanoRNAseq data

**Fig. 1:**
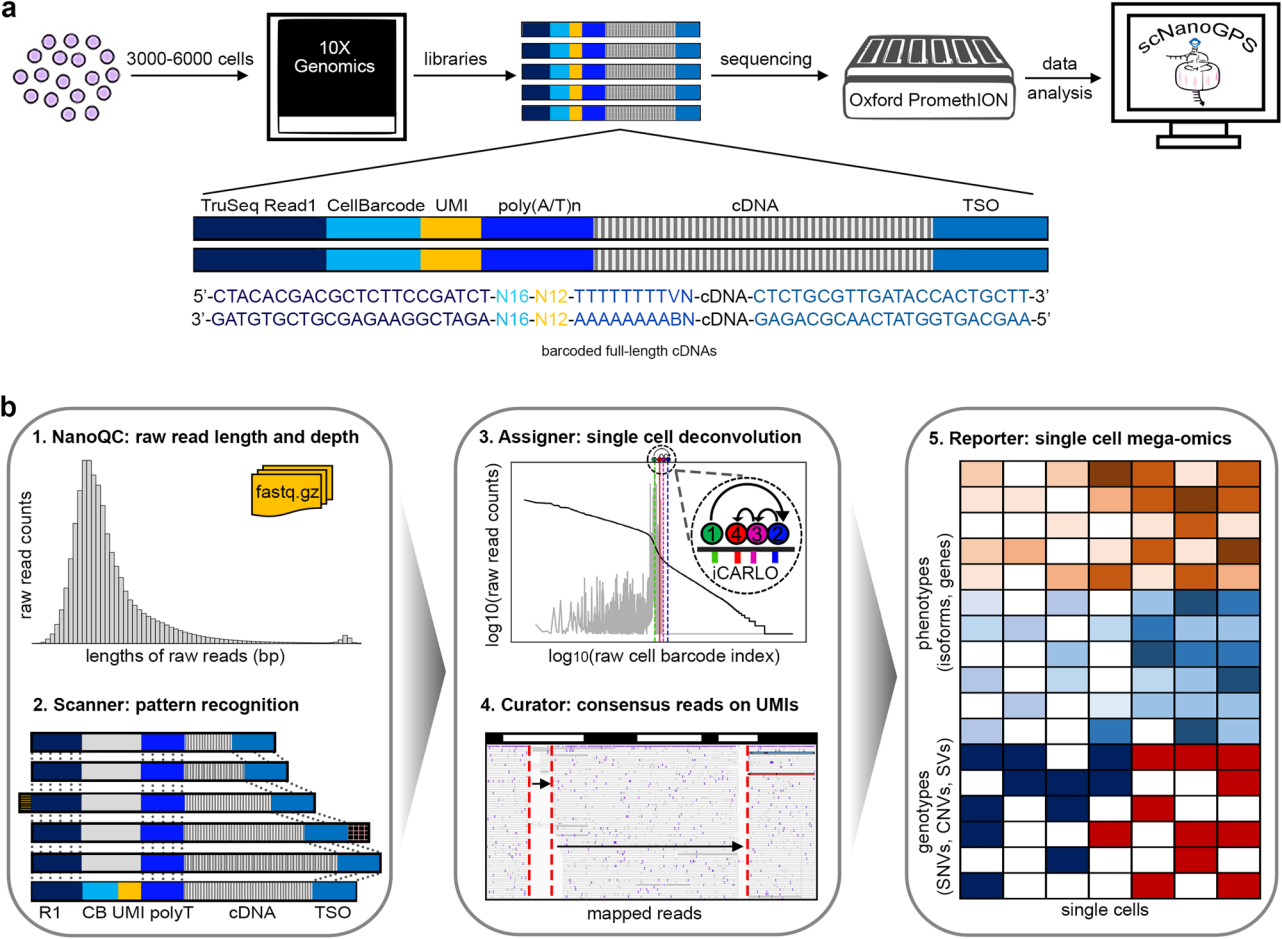
Schematic diagrams of scNanoRNAseq and scNanoGPS workflows. **a** Experimental workflow and library structure of scNanoRNAseq. **b** Computational workflow of scNanoGPS.

The high throughput scNanoRNAseq data are generated through two major steps: (i) barcoding full-length cDNAs of single cells/nuclei using high throughput droplet barcoding system (10× Genomics), (ii) performing high throughput long-read sequencing using PromethION (Oxford Nanopore Technologies) (Fig. 1a, Online Methods). Accordingly, the computational workflow of scNanoGPS begins with quality control and scanning reads that have expected patterns of adapter sequences, i.e., TruSeq R1 adapter on 5′-ends and TSO adapter on 3′-ends of raw reads (Fig. 1a, b, Fig. S1, Online Methods). Next, we developed an algorithm called **i**ntegrated **C**rude **A**nchoring and **R**efinery **L**ocal **O**ptimization (iCARLO) to detect true CBs. In this algorithm, a raw list of CBs is determined by searching for a crude anchoring point through thresholding partial derivatives of the supporting reads of individual CBs. The threshold is then extended from the crude anchoring point by an empirical percentage (10%) to rescue true CBs that have much fewer reads than others in the same experiment. Next, the CBs within two Levenshtein Distances (LDs) are curated and merged to rescue misassigned reads due to errors in CB sequences. CBs with less than 300 genes are filtered out by default. iCARLO is implemented in the “Assigner” function, which outputs a list of true CBs and then deconvolutes all qualified reads into single-cell FASTQ files.

To identify true UMIs to accurately measure gene expression levels, scNanoGPS first maps single-cell reads against reference genome GRCh38^10^ using MiniMap2^11^ (Fig. 1b). Reads that map to the same genomic regions (within <5 bp) are grouped into read clusters to achieve batch computing. All reads belonging to the same read clusters and having UMIs within two LDs are considered to be amplified from the same RNA molecules. Thus, their corresponding UMIs are curated to be identical. To overcome sequencing errors in single molecule reads with identical UMIs are collapsed into consensus sequences using SPOA^12^ for better detection of point mutations in gene bodies. scNanoGPS then re-maps single-cell consensus reads to generate single-cell consensus BAM files. Lastly, to better facilitate scNanoRNAseq data analysis, we compiled existing long-read specific tools^13–15^ into scNanoGPS to form a complete pipeline to calculate gene expression, isoform expression, and point mutation profiles of individual cells from single-cell consensus BAM files. Furthermore, single-cell copy numbers can be calculated using our previously published algorithm CopyKAT^16^. These data are being used to detect cell-type-specific isoforms and mutations as well as gene expressions to demonstrate the applications of scNanoGPS in investigating the underlying mechanisms of human tumors.

To summarize, scNanoGPS achieves complete independence through the iCARLO algorithm to detect true CBs and constructs consensus molecules from reads with the same UMIs to call mutations accurately and count RNA molecules. In contrast, both Sockeye and BLAZE^9^ rely on a barcode whitelist to detect possible CBs (Supplementary Table 1). By the time of submission, we noticed that BLAZE^9^ doesn’t support UMI detection. The UMI function in Sockeye depends on high-quality reads that filter out large portions of data. Moreover, scNanoGPS assembles variant detection pipelines together to call mutations from consensus reads. Taken together, scNanoGPS provides a full spectrum of functional modules to analyze scNanoRNAseq data from raw FASTQ data to same-cell multi-omics profiles of thousands of cells.

### Performance of scNanoGPS in processing high throughput scNanoRNAseq data

**Fig. 2:**
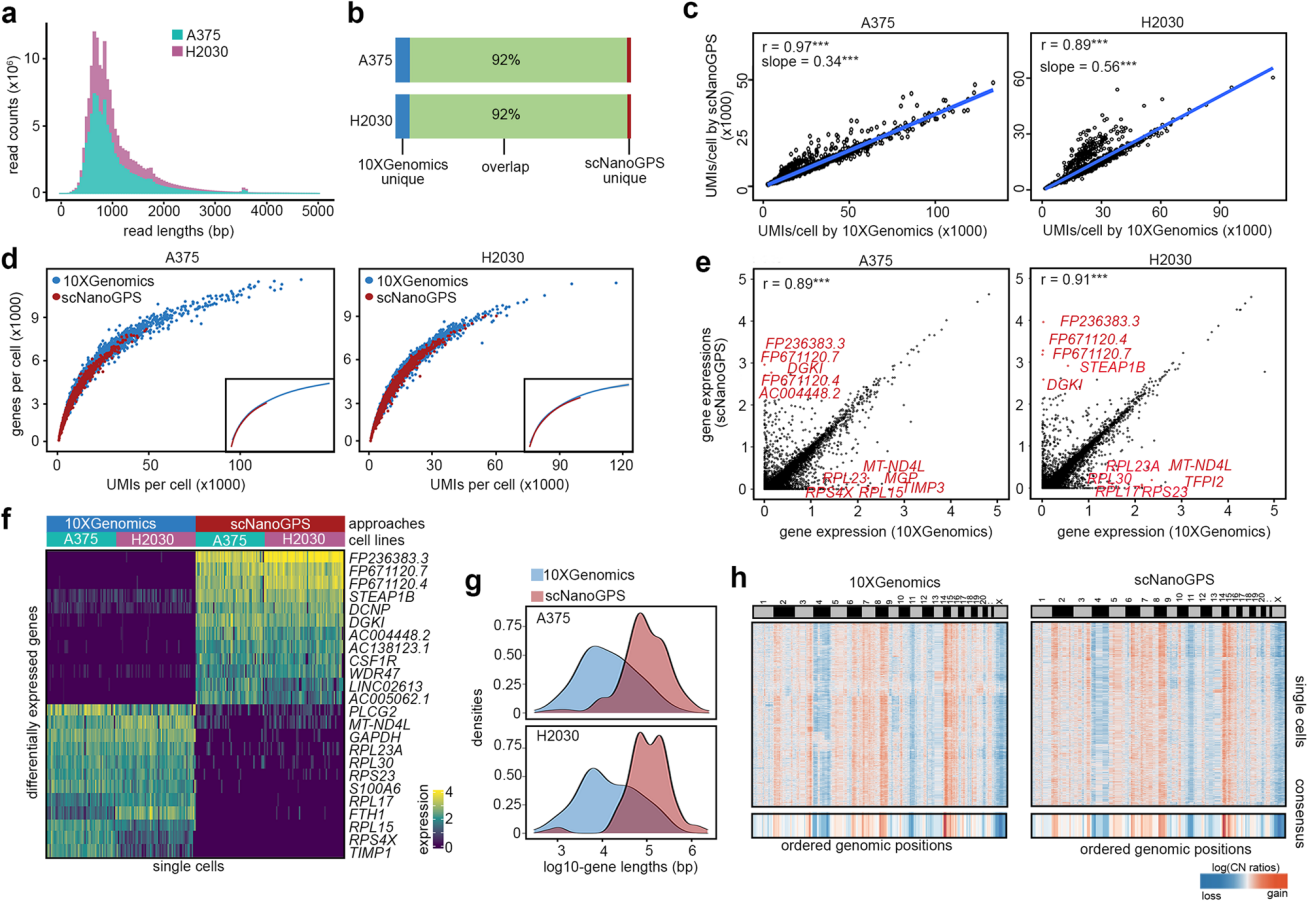
Performance of scNanoGPS in processing scNanoRNAseq data. **a** Length distribution of raw reads of two cancer cell lines. **b** Intersection of true cell barcodes detected by scNanoGPS and standard NGS approaches. **c** Pair-wised scatter plots of UMI counts per cell detected by two approaches. ***, Pearson correlation *P*-value < 2.2e−16. **d** Pair-wised scatter plots of gene detection versus UMI counts of single cells. **e** Single-cell gene expression levels calculated by two approaches. ***, Pearson correlation *P*-value < 2.2e−16. **f** Heatmap of genes with significantly different expression levels in two approaches. **g** Density plots of the length of genes with significantly different expression levels in two approaches. **h** Heatmap of single-cell copy number profiles of H2030 calculated by CopyKAT from matched single-cell transcriptomes of two approaches. Source data are provided as a Source Data file.

To test the technical performance, we applied scNanoGPS to process the scNanoRNAseq data of two cancer cell lines, A375 and H2030. Strikingly, PromethION yielded 67.4 million reads of the A375 library on one flow cell and 105.3 million reads of the H2030 library on another flow cell. This resulted in averaged depths of 15,944 reads per cell in A375 and 21,710 reads per cell in H2030, which were close to NGS scRNAseq depths (Supplementary Table 2). The median read lengths of both datasets were around 900 bp, consistent with the traces of pre-sequencing cDNAs (Fig. 2a, Fig. S2a, b). The scNanoGPS results showed that most reads (A375: 86.80%, H2030: 87.31%) had the expected pattern of adapter sequences. In total, we detected 3649 and 4212 cells with averaged coverages of 2688 and 3553 genes per cell, respectively. We generated standard NGS 3′-scRNAseq (10× Genomics) data from the same cDNA pools as previously described^5,6^. Since 3′-scRNAseq (10× Genomics) was a widely used mature method and sequenced on Illumina sequencer (NovaSeq 6000) that has very low error rates in CBs and UMIs, we treated these data as the gold standard to benchmark scNanoGPS barcode detection accuracy. Our analysis showed that scNanoGPS achieved high concordance (92%) with the standard 10× Genomics data in detecting true CBs (Fig. 2b), with minor dis-concordance in thresholding low-quality cells that could be mitigated by secondary analyses (Fig. S3a, b, Online Methods). scNanoGPS standalone achieved high accuracies in detecting true CBs, comparable to the performance of both whitelist-dependent approaches, i.e., BLAZE^9^ and Sockeye (Supplementary Table 3). We noted that without the guidance of a whitelist or known CBs, scNanoGPS took longer to scan and curate CBs. However, it consumed less memory when compared to Sockeye and BLAZE^9^ (Supplementary Table 3). Lastly, we recorded the time and space usages of each functional module of scNanoGPS when analyzing the example dataset, A375, in Supplementary Table 4 to provide guidance on the preparedness of computing resources.

Next, we compared the number of UMIs detected by scNanoGPS to standard 10× Genomics data. Our results revealed significantly high correlations (A375: Pearson’s *r* = 0.97, *P*-value < 2.2e−16; H2030: Pearson’s *r* = 0.89, *P*-value < 2.2e−16) (Fig. 2c). The gene detection rates per UMI were similar as well, although the numbers of UMIs per cell were fewer in long-read data compared to 10× Genomics data (A375: coef = 34%; H2030: coef = 56%) due to lower sequencing depths (Fig. 2c, d). The combination of CBs, UMIs, and genes detected by scNanoGPS reached up to 72% (SD: 2%) concordance on average with the NGS approach, confirming the reliability of barcoding detection results (Supplementary Table 5).

Further, we compared single-cell transcriptomes computed by scNanoGPS to standard 10× Genomics 3′-scRNAseq data. Our results again showed that scNanoGPS achieved high concordance (A375: Pearson’s *r* = 0.89, *P*-value < 2.2e−16; H2030: Pearson’s *r* = 0.91, *P*-value < 2.2e−16) in measuring gene expression levels compared to standard 10× Genomics data (Fig. 2e). Down-sampling analysis suggested that ~15,000 long-reads per cell on average are needed to robustly profile single-cell full-length transcriptomes (Fig. S4a–d). There were only small fractions (A375: 0.92%; H2030: 0.79%) of detected genes showing significantly different expression levels (FDR-adjusted *P*-values < 0.05, |log_2_(Fold Changes)| ≥ 1) between the two approaches (Fig. 2e). Of note, 10× Genomics 3′-scRNAseq showed a higher chance of detecting ribosomal genes, whereas scNanoGPS detected more long noncoding RNA (lncRNA) genes and pseudogenes (Fig. 2e-f). The lengths of scNanoGPS enriched genes were significantly longer than NGS enriched genes (Fig. 2g. A375: *P*-value = 8.16 × 10^−10^; H2030: *P*-value = 1.96 × 10^−9^), which we suspected was due to minor fragmentation bias against longer genes in NGS library preparation as previously reported^17^.

To investigate whether long-read single-cell transcriptome data could serve as a data source for inferring genome-wide DNA copy number variations (CNVs), we ran CopyKAT^16^ on H2030, which was known to have aneuploidy. As expected, our results showed that H2030 had genome-wide CNVs including amplification on Chr2, 3q, 5, 6q, 7, 8q, 14, 15p and deletions on Chr 4, 11p (Fig. 2h). The averaged pair-wised Pearson’s correlation between the two approaches reached up to 94%, confirming the feasibility of inferring CNVs using scNanoRNAseq data.

**Fig. 3:**
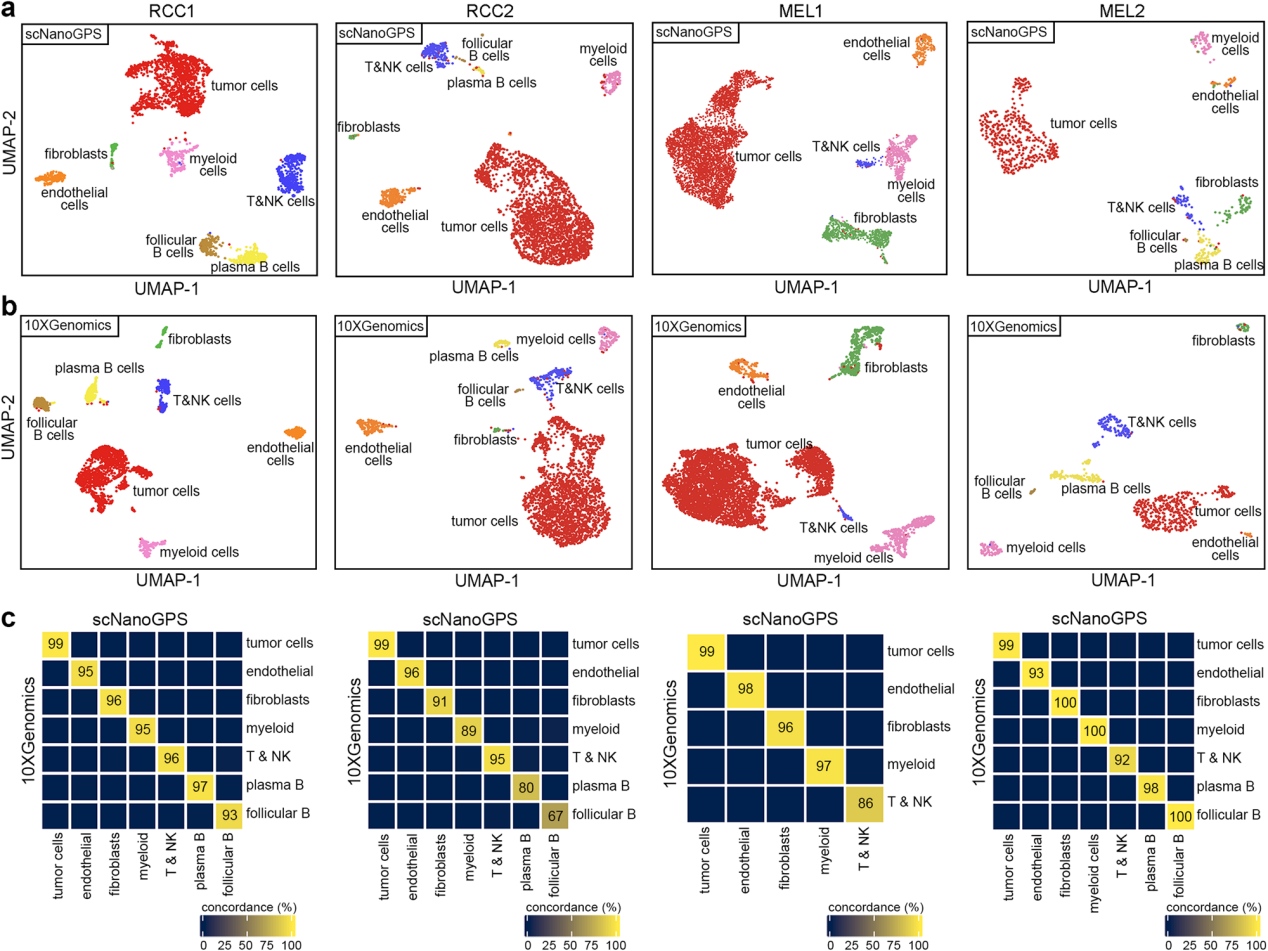
Performance of scNanoGPS in dissecting cell types in the tumor microenvironment. **a** UMAP projection of major cell types of four frozen tumors using data processed by scNanoGPS. **b** UMAP projection of major cell types of four frozen tumors using NGS data. **c** Concordance of cell typing results between scNanoGPS and NGS approaches. Only cells detected in both approaches were used for calculation.

To evaluate whether scNanoGPS can robustly detect major cell types in human tumors, we performed scNanoRNAseq on 4 frozen tumors collected from renal cell carcinoma (RCC1, RCC2) and melanoma (MEL1, MEL2) patients (Supplementary Table 2). We performed unbiased clustering of all cells within each tumor (Fig. 3a) and annotated epithelial cells having both aneuploidy and high expression levels of known cancer-type-specific genes as tumor cells (Fig. S5a, b). In consistence with previous studies^3,16,18,19^, our data showed that tumor cells outcompeted normal epithelial cells in all 4 tumors due to strong fitness advantage. The non-tumor cell-type clusters were annotated using known cell-type markers. For fair comparisons, we conducted the same clustering and annotations on paralleled 10× Genomics data (Fig. 3b). Our results confirmed high concordance between the two approaches (Fig. 3c), except that scNanoGPS rescued more lymphocytes that expressed far fewer genes than other cells in the same experiment.

To summarize, our analyses showed that scNanoGPS reliably deconvoluted long-reads into single-cells and single-molecules to detect single-cell transcriptomes and dissect the tumor microenvironment (TME) from scNanoRNAseq data.

### scNanoGPS enables the detection of cell-type-specific splicing profiles in human tumors

**Fig. 4:**
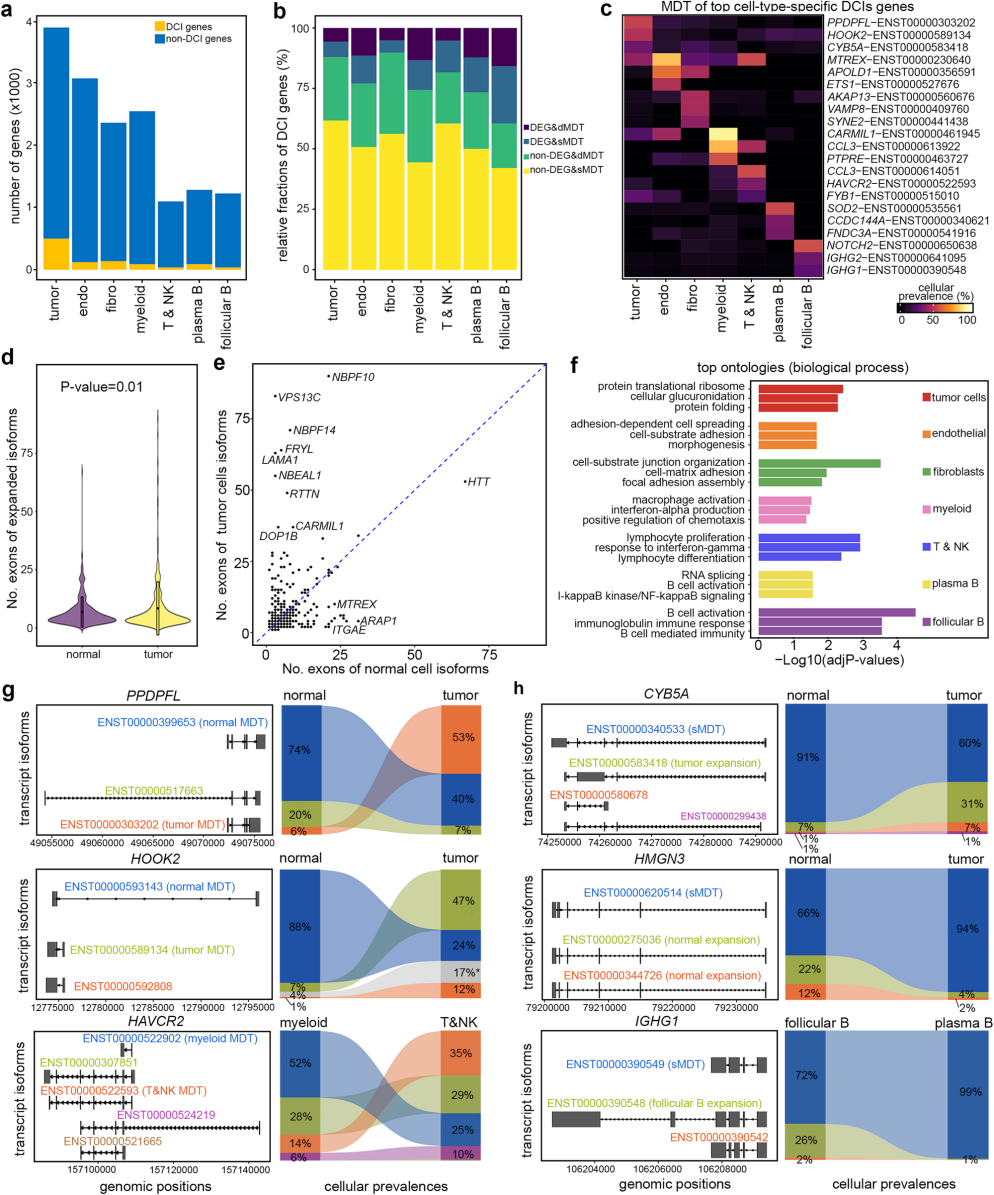
Profiling cell-type-specific isoforms in the tumor microenvironment. **a** Numbers of genes with DCIs in seven cell types of a kidney tumor. **b** Stratification of genes with DCIs based on the status of gene expression levels and most dominant transcripts (MDTs). dMDT distinct among cell types, sMDT shared across cell types. **c** Heatmap of the cellular frequencies of top cell-type-specific MDTs. **d** Violin plot of numbers of exons of tumor and normal cell preferred isoforms. *P*-value: two-sided paired *t*-test (*n* = 335). Boxes inside the violin plots are centered at the median and bounded by the first (Q1) and third quartile (Q3). **e** Pair-wised scatter plot of the numbers of exons of expressed genes in tumor and normal cells. **f** Top gene ontologies (MSigDB: GO: BP) of cell-type-specific DCI genes. *P*-values: Fisher’s Exact *t*-test, adjusted with Benjamini and Hochberg method. **g** Examples of cellular frequencies of isoforms of genes expressing different MDTs in different cell types. **h** Examples of cellular frequencies of isoforms of genes expressing the same MDTs in different cell types. Source data are provided as a Source Data file.

To assess the robustness of scNanoGPS in discovering splicing isoforms of different cell types in the TME, we performed a detailed isoform analysis of a frozen kidney tumor (RCC1). On average, we detected six transcripts per gene by referring to all known transcripts in GENCODE (*v32*)^20^. Comparisons with bulk RNAseq (NGS) data of the same tumor revealed that up to 47% isoforms were only detected by TGS approaches (Fig. S6a), while isoform expression levels showed moderate to high concordance (median: 96%; mean: 68%; Fig. S6b). Further analyses showed that each cell type tended to express a combination of multiple isoforms, consistent with a recent study that reported isoform specificity in mouse cortex^21^. To identify cell-type-specific preference of isoforms, we compared the relative compositions of different isoforms of each gene among all 7 major cell types (Online Methods). Our analysis identified 1014 genes that preferably expressed significantly different combinations of isoforms (DCIs, Chi-sq test *P*-values < 0.05 and |Prevalence Differences| ≥ 10%) among all cell types, including 499 DCI genes in tumor cells, 122 in endothelial cells, 137 in fibroblasts, 90 in myeloid cells, 38 in T & NK cells, 90 in plasma B cells and 38 in follicular B cells (Fig. 4a–c, Supplementary Data 1–7). Of note, we detected 2–4 times more genes with DCIs in tumor cells compared to immune and stromal cell types. The top-ranked tumor-cell-specific DCI genes were *PDE10A* and *NR4A2* involved in cAMP pathways. Additionally, we observed that tumor-cell-specific isoforms had slightly more exons (Fig. 4d, paired *t*-test *P*-value = 0.01), particularly in genes with more than 20 exons such as *NBPF10*, *VPS13C*, and *NBPF14* (Fig. 4e). Geneset (MSigDB, GO: BP) analysis showed that cell-type-specific DCI genes were commonly enriched in pathways related to cell-type-specific functions, such as cAMP pathway and resistance associated glucuronidation pathway in tumor cells, interferon-alpha production pathways in myeloid cells, lymphocyte proliferation pathway in lymphocytes, and immunoglobin-mediated immune responses in B cells (Fig. 4f).

Notably, we observed that a large portion (mean: 83%, SD: 9.9%) of cell-type-specific DCI genes were not detected as differentially expressed genes (DEGs) in all cell types (Fig. 4b). Tumor-cell-specific DCI genes preferably expressed different most-dominant-transcripts (MDTs)^22^ regardless of their overall gene expression levels. For instance, the proliferation gene *PPDRPFL* expressed MDTs, ENST00000303202 in tumor cells and ENST00000399653 in normal cells, although the overall expression levels of this gene in the two cell types were not significantly different (Fig. S7a, b, Fig. 4g). On the other hand, the organelle hook protein coding gene *HOOK2* preferably expressed ENST00000589134 in tumor cells and ENST00000593143 in normal cells and had significantly higher expression levels in tumor cells compared to normal cells (Fig. 4g, Fig. S7b). In addition, we observed a small fraction of cell-type-specific DCI genes that expressed the same MDTs, but their cellular fractions were different between tumor and normal cells. One example was *CYB5A* which expressed isoform ENST00000340533 in 60% of tumor cells but 91% of normal cells. Another example was *HMGN3* which expressed isoform ENST00000620514 in 94% of tumor cells but 66% of normal cells (Fig. 4h, Fig. S7c).

We also observed isoform preference in immune and stromal cell types, although fewer genes were involved compared to tumor cells. For instance, myeloid and T cells both expressed *HAVCR2*, yet the MDTs of this gene were distinct in the two immune cell types (Fig. 4g, Fig. S7b). In contrast, the B cell-specific gene, *IGHG1*, expressed the same MDT (ENST00000390549) in both follicular and plasma B cells, but the cellular prevalence of this MDT was significantly different in the two B cell subtypes (72% in follicular B cells, 99% in plasma B cells) (Fig. 4h), indicating its relevance to sub-cell-type-specific functions.

In summary, we demonstrated the usage of scNanoGPS in studying cell-type-specific splicing isoforms in tumors. Our results showed that a larger portion of genes utilized different MDTs in both tumor and immune cell types. Genes expressing the same MDTs may have distinct cellular prevalence in different cell types regardless of overall gene expression levels.

### Transcriptome-wide mutations of different cell types in the TME

**Fig. 5:**
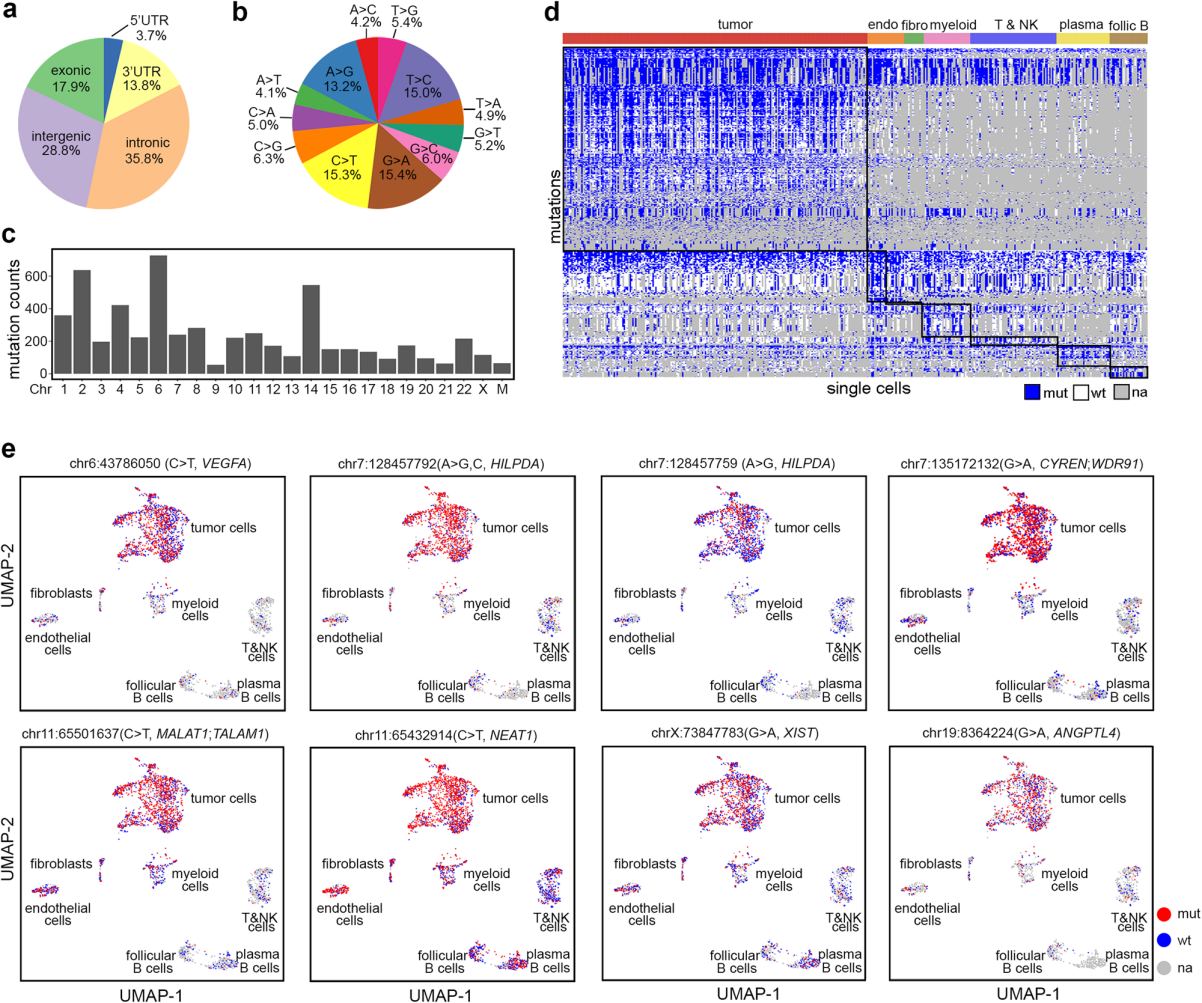
Transcriptome-wide mutation profiling of different cell types in the tumor microenvironment. **a** Pie chart of relative fractions of all mutations in different gene regions. **b** Pie chart of mutation types of all mutations. **c** Number of mutations in different chromosomes. **d** Heatmap of cell-type-specific somatic deMuts in all cells. **e** UMAP projections of single cells labeled with eight examples of tumor-cell-specific deMuts after SoupX. Source data are provided as a Source Data file.

To accurately detect transcriptome-wide mutations in single cells, we built consensus sequences of single molecules and required at least 2 consensus reads supporting variants. In addition, we filtered out mutations that were detected in less than 1% of cells over all cells or less than 5% within individual cell types (Online Methods), which removed most random errors (Supplementary Table 6). In total, we detected 6390 mutations from 3470 single nuclei transcriptomes of a frozen kidney tumor (RCC1). Further, we classified these mutations into germline mutations that were detected in >90% of cells with coverages and somatic mutations that were detected in a wide range of cells across all cell types (Fig. S8). Our analysis showed that 90.6% of germline mutations aggregated from scNanoGPS results were detected in pseudo-bulk long-read data (Fig. S9a). However, the concordance between TGS with NGS was moderate (germline: 46.5% and somatic: 39.8%), which were largely due to differences in gene body coverages, e.g., NGS bulk RNAseq data had poorer uniform gene body coverages (Fig. S9c). Among all mutations, 17.9% were in exonic regions, while others were spreading across non-coding regions (35.8% intronic, 28.8% intergenic, 3.7% 5′UTR, and 13.8% 3′UTR) (Fig. 5a). Statistical analysis revealed that the odds of mutation detection were significantly (Chi-sq *P*-values < 2.2e−16) increased in exonic (odds ratio: 7.8), 5′-UTR (odd ratio: 5.2) and 3′-UTR (odds ratio: 4.8) regions and decreased in intergenic (odds ratio: 0.65) and intronic (odds ratio: 0.46) regions as expected. Of note, we observed 53.3% of exonic mutations were nonsynonymous. Transition mutations were more frequent (13–15%) than transversion mutation types (4–6%) (Fig. 5b), consistent with a previous study^23^. The potential RNA editing (C > U) events^24^ were not distinguishable from C > T transition (15.3%). These transcriptome-wide mutations were distributed across all chromosomes except for Y-chromosome (Fig. 5c). Our data revealed 4 shared mutation hotspots on Chr2, 6, 14, and 22 in all 7 cell types, where Chr6 mutation hotspot was known to affect HLA gene clusters in both tumor and normal development^25^ and Chr14 hotspot harbored mutations in LncRNAs (Fig. S10a, b).

Our data showed that the mutated transcripts were detected in 15–25% of cells of each cell type (Fig. S8a), indicating the dependency of mutation detection rates on gene expression levels and transcript capture efficiency in single-cell RNA sequencing technology^3,26–28^. The number of total mutations in individual cells varied across cell types from 1119 SNVs in tumor cells to 758 in endothelial cells, 506 in fibroblasts, 703 in myeloid, 461 in lymphoid, 484 in plasma cells, and 501 in follicular B cells on average (Fig. S11). To mitigate the false positive detection of mutations due to ambient RNAs, we ran SoupX^29^ to de-noise the data and removed mutations that either landed on ambient RNAs or mapped to non-coding regions (Fig. S12). Next, we compared the cellular frequencies of mutations among different cell types to identify mutations that were differentially expanded (deMuts). Our results showed that tumor cells had the largest number (*N* = 609) of deMuts, followed by myeloid cells (*N* = 99), plasma B cells (*N* = 63), endothelial cells (*N* = 57), follicular B (*N* = 29), lymphocytes (*N* = 26), and fibroblasts (*N* = 1) (Fig. 5d, Supplementary Data 8–14). Observation of lower mutation burden in normal cell types is consistent with prior knowledge of spontaneous mutation accumulation in normal organs throughout life time^30^. Tumor-cell-specific deMuts included many COSMIC genes, such as *VEGFA, NEAT1, MALAT1, HILPDA*, etc. (Fig. 5e). Further, we detected several genes that were both mutated and differentially spliced in the same cell types, such as *HMGN3* and *UBE2G2* in tumor cells, *CD74* and *IFI30* in myeloid cells, and *RPS2* in endothelial cells indicating their important roles in tumorigenicity. Additionally, we observed several cell-type-specific deMuts involved in the spliceosome gene set (GSEA: KEGG pathway), such as *SRSF3* and *HNRNPC* mutants in tumor cells and *DDX5* mutants in endothelial cells (Fig. S11b).

In summary, we demonstrated that scNanoGPS could robustly detect cell-type and cell-state-specific mutations. Our data highlighted the importance of identifying population-specific mutations to understand their functional roles in cancer progression.

## Discussion

In this study, we develop a computational tool called scNanoGPS to facilitate high throughput single-cell long-read sequencing data analysis. scNanoGPS achieves independent deconvolution of raw data without the guidance of short-reads or barcode whitelists and calculates genotypes-phenotypes of thousands of individual cells, addressing the major computational challenges of the emerging scNanoRNAseq technology.

Two previous methods, called Sicelore^5^ and scNapBar^8^, are developed to deconvolute raw reads. However, both rely on the guidance of paralleled NGS data. An experimental method called scCOLOR-seq^7^ is developed to reduce error rates in barcodes by designing bi-nucleotide repeats in barcode sequences^7^, but it relies on prior knowledge of true barcodes to curate errors and requires customized synthesis of gel-beads to adapt to the droplet system. Two other NGS-independent barcode deconvolution tools, Sockeye and BLAZE^9^ rely on the comparison of long-reads data with a barcode whitelist. This is worrisome when barcode manufacturing products deviate from theoretic lists, or the wrong version of the whitelist is used. In comparison, scNanoGPS achieves high accuracy in detecting true CBs directly from long-read data using a multi-step algorithm, iCARLO, without the guidance of NGS data or barcode whitelist.

Dysregulation of transcript isoforms plays a critical role in tumor progression^22,31–33^. Previous studies show that only ~20–40% of transcriptional isoforms could be reconstructed from NGS bulk RNAseq data^34–36^. Long-read single-cell sequencing technologies enable in-depth annotation of splicing isoforms at single-cell levels. scNanoGPS provides a robust computational tool to achieve this goal. Our analysis of a frozen kidney tumor reveals that all major cell types in the tumor commonly express a combination of different isoforms instead of one canonical isoform. Another important finding is that tumor cells preferably express different MDTs of tumor suppressors, although their overall gene expression levels are not significantly different from normal cells. Our results imply that the discovery of cancer-specific genes using gene expression levels may only be revealing the tip of the iceberg of transcriptional diversities in cancer.

scNanoGPS provides a powerful approach for synchronic tracing of cell lineage and cell fate by measuring both plastic phenotypic markers (genes, isoforms) and stable genetic markers (mutations, copy numbers) of the same cells to study tumor evolution and therapeutic responses. scNanoGPS detects transcriptome-wide point mutations with accuracy by building consensus sequences of single molecules and performing consensus filtering of cellular prevalence, which removes most false calls due to random sequencing errors. However, our consensus approach does not address errors in calling small indels that represent the major type of Nanopore sequencing errors. We expect that later versions of sequencing chemistry and nucleotide calling algorithms could address this limitation.

One potential confounding factor of our analyses would be the false discoveries associated with ambient RNAs that may not be fully addressed by SoupX^29^; particularly, it’s challenging to correct the bias in isoform quantifications due to ambient RNAs in low-quality samples.

In addition to what we have demonstrated in this study, scNanoGPS has broad applications in many other genomic research areas, such as measuring single-cell gene fusions, tandem repeats, splicing velocities, repetitive genes, or long non-coding genes to investigate diverse mechanisms of human diseases including but not limited to cancer.

## Methods

### Cancer cell line and tumor tissue samples

All research activities of this study comply with relevant ethical regulations of Northwestern University Institutional Biomedical Review Board. The A375 cell line was provided by Dr. Michael A. Davies at MD Anderson Cancer Center, and H2030 was obtained from the Antibody and Bioresource Core Facility at Memorial Sloan Kettering. The cell lines are standard commercialized cancer lines. The four frozen tumors (RCC1, RCC2, MEL1, MEL2) were obtained from UT MD Anderson Cancer Center. The tumor tissues were collected with written consent under IRB approval at UT MD Anderson Cancer Center. All materials were transferred under the approval of the Material Transfer Agreement between institutions.

### Preparation of single nucleus suspension

Single nucleus suspensions of two cancer cell lines were prepared by following the 10X Genomics protocol (CG000365 Rev C). Single nucleus suspensions of frozen tumor tissues were prepared according to the method as previously described^27^. Frozen tissue was cut into tiny pieces in a 10-cm Petri dish with 500 µl-2 ml NST-DAPI buffer for 10-15 minutes and filtered through a 40 mm Flowmi into 1.5 ml LowBind Eppendorf tube and centrifuged at 4 °C 300*g* for 5 min. The resulting nuclei pellet was washed three times with cold Nuclei Wash and Resuspension Buffer. After cell counting, the nuclear suspension was centrifuged again and resuspended in the appropriate volume depending on the nuclei counting results. Preparation of NST-DAPI buffer: Mix 800 ml of NST solution (146 mM NaCl, 10 mM Tris-base (pH 7.8), 1 mM CaCl_2_, 21 mM MgCl_2_, 0.05% (wt/vol) BSA and 0.2% (v/v) Nonidet P-40) with 200 ml of DAPI solution (106 mM MgCl_2_ and 10 mg of DAPI). The solution is filter-sterilized and is stored at 4 °C in the dark for up to 1 year. Preparation of Nuclei Wash and Resuspension Buffer: 1× PBS with 1.0% BSA and 0.2 U/μl RNase Inhibitor.

### Preparation of barcoded full-length cDNAs of single nuclei

Single nucleus suspensions were loaded onto 10× Genomics Chromium Controller (iX) with Chip J to capture 3000–6000 single nuclei. The full-length mRNAs and/or pre-mRNAs were barcoded with cell barcodes (CBs) and unique molecular identifiers (UMIs) through cDNA amplification using 10× Genomics protocol. We modified the cDNA amplification protocol by extending the elongation time to 3 min to enrich longer molecules as previously described^5^.

The barcoded full-length cDNA transcripts (10 ng) were amplified for five cycles with the following two customized primers synthesized by Integrated DNA Technologies (Coralville, IA): 1) 5′-biotinylated TruSeq Read 1 forward primer 5′-/5Biosg/AA AAA CTA CAC GAC GCT CTT CCG ATC T -3′ (25 nM); 2) 3′ partial TSO reverse primer 5′-NNN AAG CAG TGG TAT CAA CGC AGA GTA CAT-3′. The amplified single-cell cDNA transcripts were subjected to 0.8× SPRIselect reagent (Beckman Coulter, CA) clean-up to remove unbound and excess biotinylated primers, where the bound cDNA was eluted off the bead matrix in 45 µL of Qiagen Buffer EB (Qiagen; Valencia, CA). The eluted cDNA was further purified through the binding of the biotinylated template to Dynabeads™ M-270 Streptavidin beads (Invitrogen; Waltham, MA). Prior to the selection of the cDNA template, 15 µL of the Dynabeads™ M-270 Streptavidin beads were washed in 1 mL of 1× SSPE solution (UltraPure™ 20× SSPE Buffer (Invitrogen) freshly prepared with nuclease-free water). A magnetic stand was used to separate the streptavidin beads from the initial wash solution. The streptavidin beads were then washed three times with 15 µL of 1× SSPE with removal from the magnet and resuspension of the beads in a fresh wash solution with each repeat wash. Following the final wash, the streptavidin beads were resuspended in 10 µL of 5× SSPE solution, and the biotinylated cDNA template was added to the washed beads. This mixture was placed on a tube rotator at room temperature for 15 minutes. Post incubation on the rotator, with the biotinylated cDNA template bound to the washed streptavidin beads, the sample was placed back on the magnetic stand for separation. The cDNA-bound beads were washed twice with 100 µL 1× SSPE solution and a final wash with 100 µL Buffer EB. The use of the biotinylated forward primer and subsequent purification with the Dynabeads™ M-270 Streptavidin beads allowed for the selective depletion of cDNA missing the terminal poly(A)/poly(T) tail.

The streptavidin beads containing bound biotinylated cDNA were then resuspended in 100 µL of PCR master mix for a secondary amplification for five cycles with regular PCR primers: (1) TruSeq read 1 forward primer 5′-NNN CTA CAC GAC GCT CTT CCG ATC T-3′ and 3′ partial TSO reverse primer 5′-NNN AAG CAG TGG TAT CAA CGC AGA GTA CAT-3′. The PCR amplified product was purified with 0.8× SPRIselect reagent into a final elution of 51 µL in Buffer EB to allow for adequate template/volume for the necessary assessment of quality control (QC) metrics and PromethION library preparation.

The KAPA Biosystems HiFi HotStart PCR Kit (Roche; Basel, Switzerland) was used to prepare all PCR amplification mixtures for Nanopore library preparations. The following PCR conditions were followed for amplification: initial denaturation, 3 min at 95 °C; 5 cycles of denaturation for 30 s at 98 °C, annealing for 15 s at 64 °C, and extension for 5 min at 72 °C; followed by a final extension for 10 min at 72 °C.

### Nanopore sequencing library preparation for full-length cDNAs

Based on the sample molarity and average cDNA transcript length derived from the QC metrics, the sample input volume was calculated and used to progress into nanopore library preparation. The SQK-LSK110 ligation sequencing kit (Oxford Nanopore) was used to generate PromethION long-read cDNA libraries. The final sequencing was run on PromethION flow cell (*v9.4.2*) with one sample per flow cell by the DNA technology core at UC Davis. The output data was base-called live during the run using base-caller guppy (*v5.0.12*) in a super-accurate base-calling model.

### Generating benchmarking data with paralleled NGS sequencing of fragmentized cDNAs

The same aliquot (25% volume) of barcoded full-length cDNAs was fragmented and subjected to next-generation sequencing library preparation by following the 10× Genomics Next GEM Single Cell Gene Expression protocol. The final libraries were sequenced on the Illumina Novaseq 6000 sequencer at the NUcore at Northwestern University. The sequencing data were processed using 10X Genomics software CellRanger ARC (*v2.0*)^28^.

### QC of scNanoRNAseq data

The raw FASTQ files were processed by the scNanoGPS ‘NanoQC’ function to scan the distribution of raw read lengths, which generated a PNG plot named ‘read_length.png’ and a tab-separated table named ‘read_length.tsv’. The first and last 100 nucleotides of raw reads were extracted for sequencing quality analysis with FastQC (*v0.12.0*)^37^.

### Scanning long-reads with expected adapter patterns

The raw FASTQ files were processed by scNanoGPS ‘Scanner’ function to scan the expected adapter patterns using the parameters (match: 2, mismatch: −3, gap opening: −5, gap extension: −2, sequence identity $\ge$ 70%) equivalent to NCBI Basic Local Alignment Tool (BLAST) algorithm. Raw reads with an insert length of less than 200 bp was excluded from scanning. After ‘Scanner’, a compressed file containing parsed raw CBs named ‘barcode_list.tsv.gz’ and a filtered read sequence file named ‘processed.fastq.gz’ were generated.

### Deconvolution of long-reads into single cells

The raw reads that passed QC and pattern filtering steps were demultiplexed into single cells using scNanoGPS ‘Assigner’ function. Another input was the parsed barcode lists generated by ‘Scanner’. The true list of CBs was retrieved through an integrated algorithm called iCARLO. This algorithm included 4 steps. First, all CBs were ordered decreasingly by the number of supporting reads. The number of supporting reads and the order index were transformed into a log10 scale (Fig. 1b step 3 Assigner). The raw list of true CBs was estimated by thresholding the maximal partial derivatives of supporting reads against the rank of CBs. To buffer the changes, we smoothed the partial derivatives within each 0.001 window of log10-scaled CB ranks.

Let $X$ be the number of supporting reads of CBs, $i$ be the rank order of all CBs, and $w$ be the 0.001 smoothing windows as defined in Eq. (1).

1 $$\forall X,\,i,\,w\in \left(1,\,2,\,3,\ldots,\,N\right)$$

Each window $w$ contains a set of CBs, allowing empty, according to their rank order $i$ in log10 scale per 0.001 tick as shown in Eq. (2).

2 $$0.001\times w \, < \,{\log }_{10}i \, < \,0.001\times (w+1)$$

The partial derivatives were calculated for each CB and then smoothed by taking a median average of all values within each window $w$ as shown in Eq. (3). The crude anchoring point was defined as a threshold cutoff where a smoothing window $w$ had maximal partial derivative. We defined the raw number of CBs (*Cr*) at this crude anchoring point as follows:

3 $$Cr={10}^{{0.001\times {\arg }}_{w}\max {{med}}_{i}\left[\frac{{\log }_{10}{X}_{i+1}-{\log }_{10}X}{{\log }_{10}\left(i+1\right)-{\log }_{10}i}\right]}$$

We then extended this crude anchoring point by an empirical percentage (10%) to an external boundary (*N* = *Cr*_2_) of true CBs to rescue as many CBs as possible. In the third step, we exhaustively computed pair-wised LD distances of all *Cr*_2_ CBs. CBs within 2 LD were merged back one directionally to the CB harboring more supporting reads and obtained a collapsed list of true CBs (*N* = *Cr*_3_). Lastly, we retrieved the final list of true CBs (*N* = *Cr*_4_) by removing CBs that cover less than 300 genes. According to this final list of true CBs, the master FASTQ file resulting from ‘Scanner’ was split into single-cell FASTQ files stored in a temporal folder holding all the meta files for further usage.

### Curation of sequencing errors in single molecules

Detection of the true list of UMIs and curation of sequencing errors in single molecules was performed by using scNanoGPS ‘Curator’ function. To detect true UMIs, deconvolution of the single-cell FASTQ files was first aligned to the reference genome (GRCh38) using MiniMap2(*v2.26*)^11^ under the splicing mode (-ax splice). Reads mapped to the same genomic regions (coordinates within 5 bp) were grouped into batches. The batch calculation was conducted by paralleled computing cores. For the reads that belong to the same clusters and have UMI within two LDs were considered reads of the same molecules. These UMI sequences were curated to be identical. To curate errors in gene bodies, the reads with the same curated UMIs were collapsed into consensus sequences of single molecules using SPOA(*v.4.0.7*)^12^. Finally, the consensus reads were re-mapped against the reference genome (GRCh38) using MiniMap2^11^ under splicing mode (-ax splice) to generate consensus BAM files for all downstream analyses.

### Benchmarking CB detection function of scNanoGPS with BLAZE and Sockeye

We compared CBs detection results of scNanoGPS to BLAZE^9^ (*v1.1.0*) and Sockeye (*v0.4.0*). To evaluate the accuracies of CB detection results, the CB list extracted from matched 3’scRNAseq data with CellRanger ARC (v2.0) (10X Genomics) short read sample with CellRanger was treated as ground truth. The FASTQ files of scNanoRNAseq data of two cell lines (A375, H2030) were used as input for scNanoGPS, BLAZE, and Sockeye. To run BLAZE and Sockeye, we downloaded the right CB whitelist version (‘737k-arc-v1.txt.gz’) from the 10× Genomics website and used default parameters (2-LD) to run both tools. The CB lists extracted from scNanoGPS, BLAZE, and Sockeye were then compared to the CB list extracted from Cellranger ARC. The CBs existed in the CellRanger list were treated as ‘true positive’ detection, whereas CBs that were not extracted by CellRanger were considered as ‘false positive’ detection. The computing time and memory usage in CB detection steps were also recorded accordingly, except Sockeye, which does not execute this step separately.

### Calculation of same-cell multi-omics from consensus reads

The consensus BAM files of single cells were used as input to calculate single-cell transcriptomes, isoforms, and point mutations using scNanoGPS ‘Reporter’ functions. The single-cell gene expression profiles were calculated using FeatureCount^13^ from the Subread package^38,39^ that supports long-read gene level counting. The single-cell isoforms were calculated using LIQA^14^, a method designed to calculate isoforms from long-read data of spliced mRNAs. We used default parameters (weight of bias correction: 1, maximal distance: 20 bp) when running LIQA. The single-cell point mutation detection was conducted using a robust long-read variant detection tool, LongShot^15^. We benchmarked the LongShot results in terms of the number of SNVs with different cell prevalence and different numbers of supporting reads (Suppl. Table 1). With the elbow method, we required that each alternative allele should be supported by at least two consensus reads. The resulting VCF files of single cells were merged using BCFtools (*v1.15*)^40^. A final list of mutations of all cells was obtained by consensus filtering, where all variants detected in less than 1% of the cells were considered random errors and removed from the analysis. To differentiate between true wild types and missing values, we re-scanned the read depths of all loci in the final list by Samtools (*v1.15*)^40^. Loci with 0/0 genotypes with supporting reads >0 were defined as true wild types, otherwise, as missing values if no supporting reads were found. The final mutation loci were annotated using ANNOVAR^41^, which included dbSNP (*v150*)^42^ and COSMIC (*v96*)^43^ as references. The single-cell copy numbers were calculated using our previously published method, ‘CopyKAT’ (*v1.0.6*)^16^, with default parameters. In all final output matrices, features/genes were put in rows, while CBs were in columns.

### Single-cell gene expression data analysis: QC and defining major cell types

The gene expression matrices of NGS-based 3’-scRNAseq data were processed using CellRanger ARC^28^ (10X Genomics) and sent for downstream analysis using the ‘Seurat’ R package (*v4.1.1*)^44^. In 4 tumor samples, doublets were removed using R package ‘DoubletFinder’ (*v2.0.3*)^45^ with assumed doublet rates of 0.8% per 1000 cells. Cells with more than 10,000 genes, or more than 100,000 UMIs, considered as doublets, were removed as outliers and suspected doublets. Cells with less than 300 genes were filtered out due to low gene coverage. Furthermore, cells with higher fractions of mitochondrial genes were filtered out using arbitrary outlier cutoffs (5% in RCC1 and RCC2; 20% in MEL1 and MEL2). UMI count matrices were normalized using the ‘LogNormalize’ method in the ‘NormalizeData’ function and scaled across cells using the ‘ScaleData’ function in ‘Seurat’. The top 2000 highly variable genes were selected with the ‘FindVariableFeatures’ function based on ‘vst’ method and used for Principal Component Analysis (PCA). Next, we performed PCA and uniform manifold approximation and projection (UMAP) for dimension reduction with the top 30 PCs. ‘FindNeighbors’ function based on the top 30 PCs and ‘FindClusters’ functions were applied to perform unbiased clustering of cells. In all the samples, we defined a cluster of low-quality cells that did not express known cell type markers and had much fewer genes and UMIs compared to other cells in the same experiments. Final clustering analyses were reperformed without these low-quality cells. To identify the tumor cells, we used the UMI count matrix as input to infer chromosomal CNA profiles using the R package ‘CopyKAT’ (*v1.0.6*)^16^. Cells with genome-wide CNAs were labeled as tumor cells.

### Analyses of cell-type-specific splicing isoforms

Cell-type-specific splicing isoform analyses started from single-cell isoform expression matrices that summarized the expression levels of all known isoforms based on GENCODE (*v32*)^20^ in single cells. For pairwise two-group (cell type) comparisons, we filtered out sporadically expressed genes, which were detected in less than 5% of cells in both comparison groups. Additionally, genes with only one isoform were excluded from this analysis. To mitigate false discoveries driven by dropouts, the isoform expression levels of a given gene were aggregated across all cells within each comparison group. The aggregated counts of expressed molecules of all isoforms of a given gene in both comparison groups were sent for Chi-square tests to test whether the relative composition of different isoforms of a given gene was significantly different in the two comparison groups or not. *P*-values were adjusted using Benjamin–Hochberg (BH) correction for multiple testing with a 5% false discovery rate. The relative frequency of all isoforms of a given gene and the differences in the two comparison groups were also calculated. Finally, the genes expressing significantly different combinations of isoforms (DCIs) were defined as having FDR-adjusted. *P*-values < 0.05, and at least one isoform had different cellular prevalence in two comparison groups ≥10%. Gene ontology (GO) biological process (BP) enrichment analysis of each gene module was performed using DAVID (https://david.ncifcrf.gov).

### Detection of cell-type-specific SNVs

To further remove random errors, we filtered out the called positions that were detected in less than 5% of cells in each cell type or in comparing groups. Next, we generated a count matrix that included the number of wild-type cells (expressed only reference alleles) and the number of mutated cells (expressed variant alleles) of all candidate SNVs in both groups that were under comparison. We performed a Chi-square test to measure whether a candidate SNV had significantly different cellular frequencies. We adjusted *P*-values using BH correction for multiple testing with a false discovery rate of 5%. This analysis was only performed using data from cells that had read coverages. Cells without read coverages were not included in calculating the cellular frequencies of mutations. We determined the candidate SNVs with FDR-adjusted *P*-value < 0.05 and the differences in cellular frequencies > 0.1 as population-specific differentially expanded SNVs (deMuts).

### Summary of statistical methods

We applied Chi-sq tests to compare the relative frequencies of isoforms or mutations in two comparison groups. *P*-values were adjusted using the BH method to adjust for multiple test errors with a false discovery rate of 5%. We applied Pearson’s correlation and *P*-value to measure the similarity of gene expression profiles and the total counts of UMIs per cell between scNanoGPS and NGS approaches. A paired-two-sided *t*-test was performed to compare the number of exons of different isoforms of the same genes between tumor and normal cells. All significance cutoffs used in this study were set at 0.05.

### Reporting summary

Further information on research design is available in the Nature Portfolio Reporting Summary linked to this article.

## Supplementary information

Supplementary Information

Description of Additional Supplementary Files

Supplementary Dataset 1–14

Reporting Summary

## Data Availability

The raw sequencing data and processed data generated in this study have been deposited in the Gene Expression Omnibus (GEO) database under accession code GSE212945. Source data are provided in this paper.

## Source data

Source Data

## Code availability

Software is available at GitHub [https://github.com/gaolabtools/scNanoGPS]^46^. Codes of statistical tests are provided in the Supplementary Note.
