# Supplementary material for: High throughput single cell long-read sequencing analyses of same-cell genotypes and phenotypes in human tumors
Source: Nat Commun. 2023 Jul 11;14:4124. doi: 10.1038/s41467-023-39813-7 (PMC10336110; doi:10.1038/s41467-023-39813-7)
Supplement: Supplementary file 1 — Supplementary Information [file 41467_2023_39813_MOESM1_ESM.pdf]

Supplementary Information
Shiau, Lu, Kieser et al., 2023

Table of Contents

Figure S1 – Illustration of scNanoGPS methods, related to Figure 1

Figure S2 – Size distributions of cDNAs, related to Figure 2

Figure S3 – Identification of low-quality cells, related to Figure 2

Figure S4 – Saturation analysis of scNanoRNAseq depths in A375 and H2030 cell lines, related to Figure 2

Figure S5 – Classification of tumor and normal cells in tumor samples, related to Figure 3

Figure S6 – Comparison of isoforms detected in long and short-read sequencing data of a frozen kidney tumor RCC1, related to Figure 4

Figure S7 – Cell type specific genes with DCIs, related to Figure 4

Figure S8 – Mutation detection efficiency and frequencies in different cell types in a frozen kidney tumor RCC1, related to Figure 5

Figure S9 – Comparison of point mutations detected in long and short-read sequencing data of a frozen kidney tumor RCC1, related to Figure 5

Figure S10 – Shared mutation hotspots in all major cell types from a frozen kidney tumor RCC1, related to Figure 5.

Figure S11 – Single cell transcriptome-wide mutation profiles in a frozen kidney tumor RCC1, related to Figure 5

Figure S12 – Examples of tumor-cell-specific deMuts in single cells of a frozen kidney tumor RCC1, related to Figure 5

Table S1 - Comparison of functional modules of scNanoGPS with existing tools

Table S2 - Sample quality metrics

Table S3 - Comparison of CB detection results of three tools

Table S4 - Usages of computing resources of analyzing A375 data with scNanoGPS

Table S5 - Concordance of combinations of CBs, UMIs and genes of scNanoGPS results with CellRanger

Table S6 - Consensus filtering of RCC1 variants

Supplementary Note 1 - Codes of statistical tests

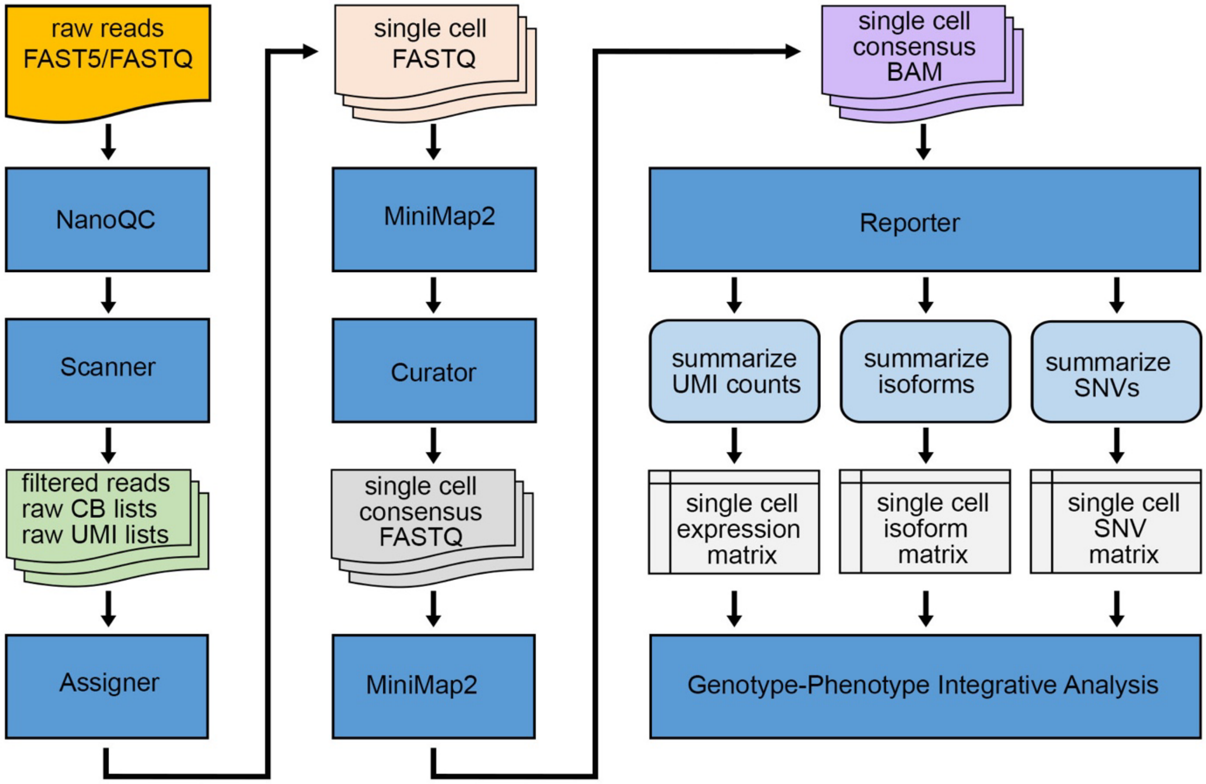

Figure S1 – Illustration of scNanoGPS methods, related to Figure 1
Blue boxes represent execution items, while others indicate files.

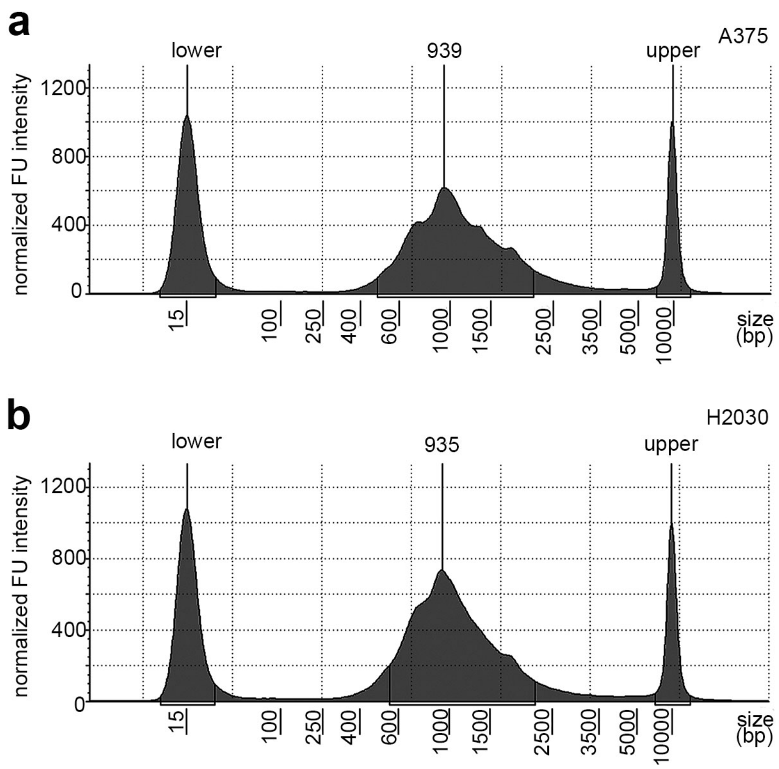

Figure S2 – Size distributions of cDNAs, related to Figure 2
The TapeStation traces of full-length cDNAs of **a**, A375 and **b**, H2030 before making sequencing libraries.

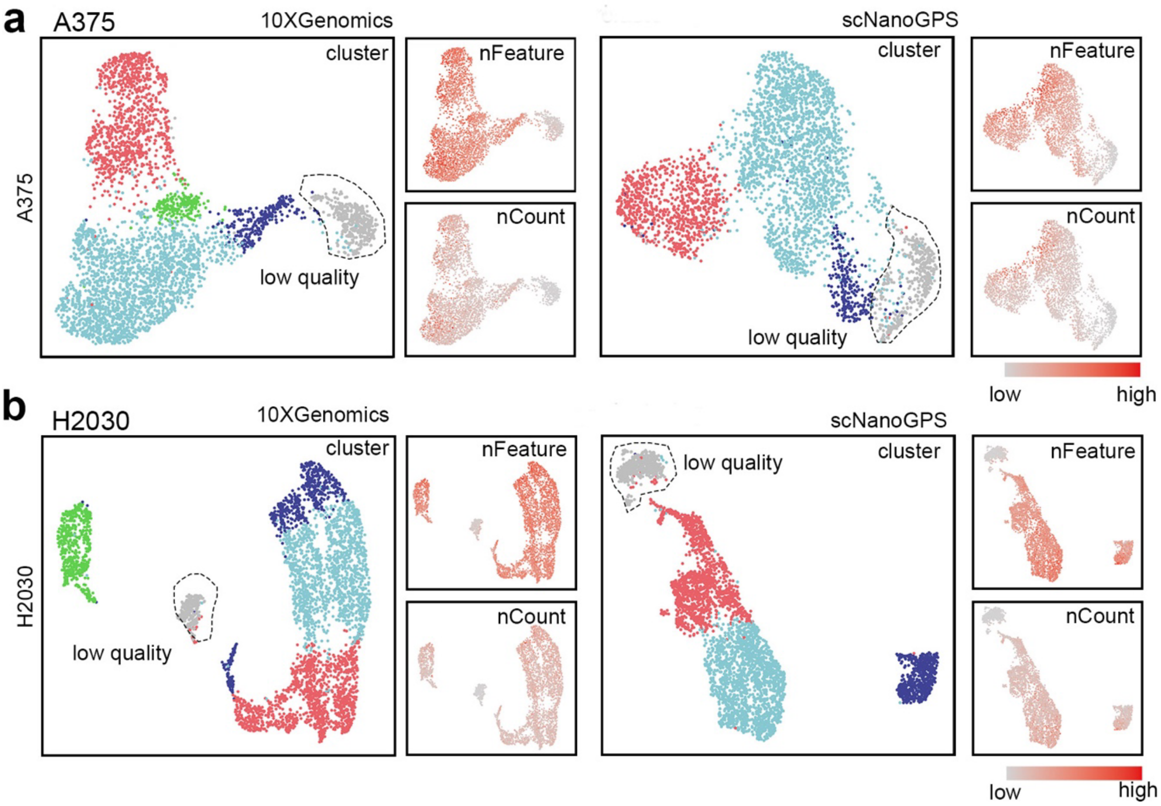

Figure S3 ─ Identification of low-quality cells, related to Figure 2

a, UMAPs of low-quality cells detected by NGS and scNanoGPS in A375. **b**, UMAPs of low-quality cells detected by NGS and scNanoGPS in H2030.

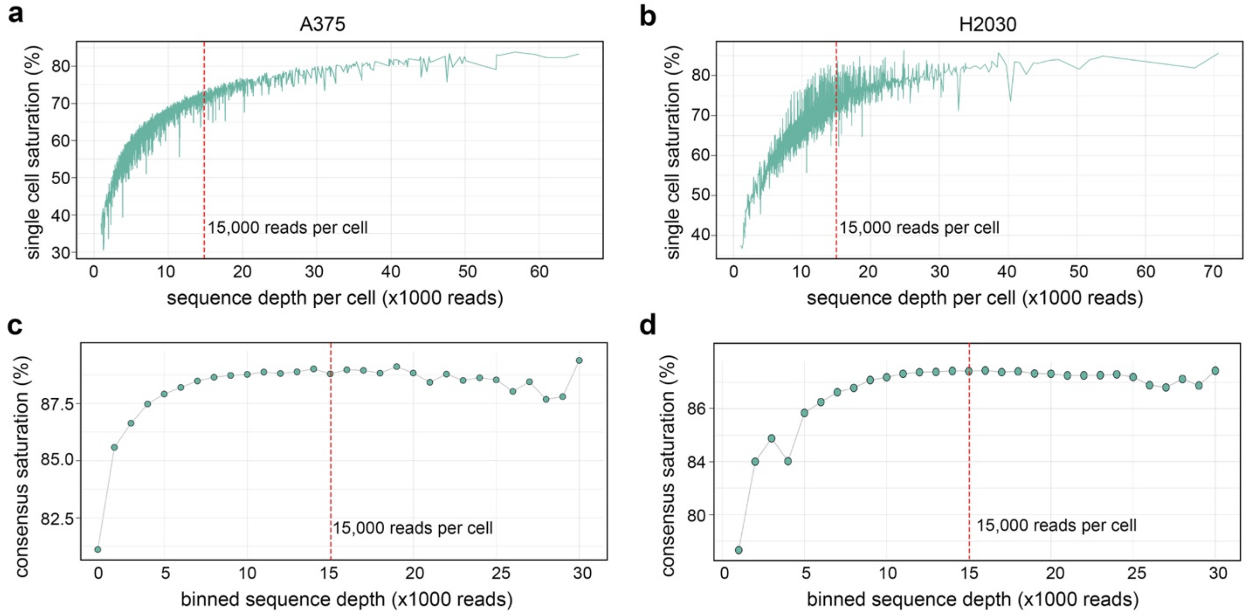

Figure S4 – Saturation analysis of scNanoRNAseq depths in A375 and H2030 cell lines, related to Figure 2
Pearson's correlation of single cell transcriptome profiles with ground truth (NGS 3'scRNAseq consensus profile) of A375 (**a**) and H2030 (**b**). Pearson's correlation of mini-bulk (averages of single cells within binned read depths) long read transcriptomes with ground truth of A375 (**c**) and H2030 (**d**).

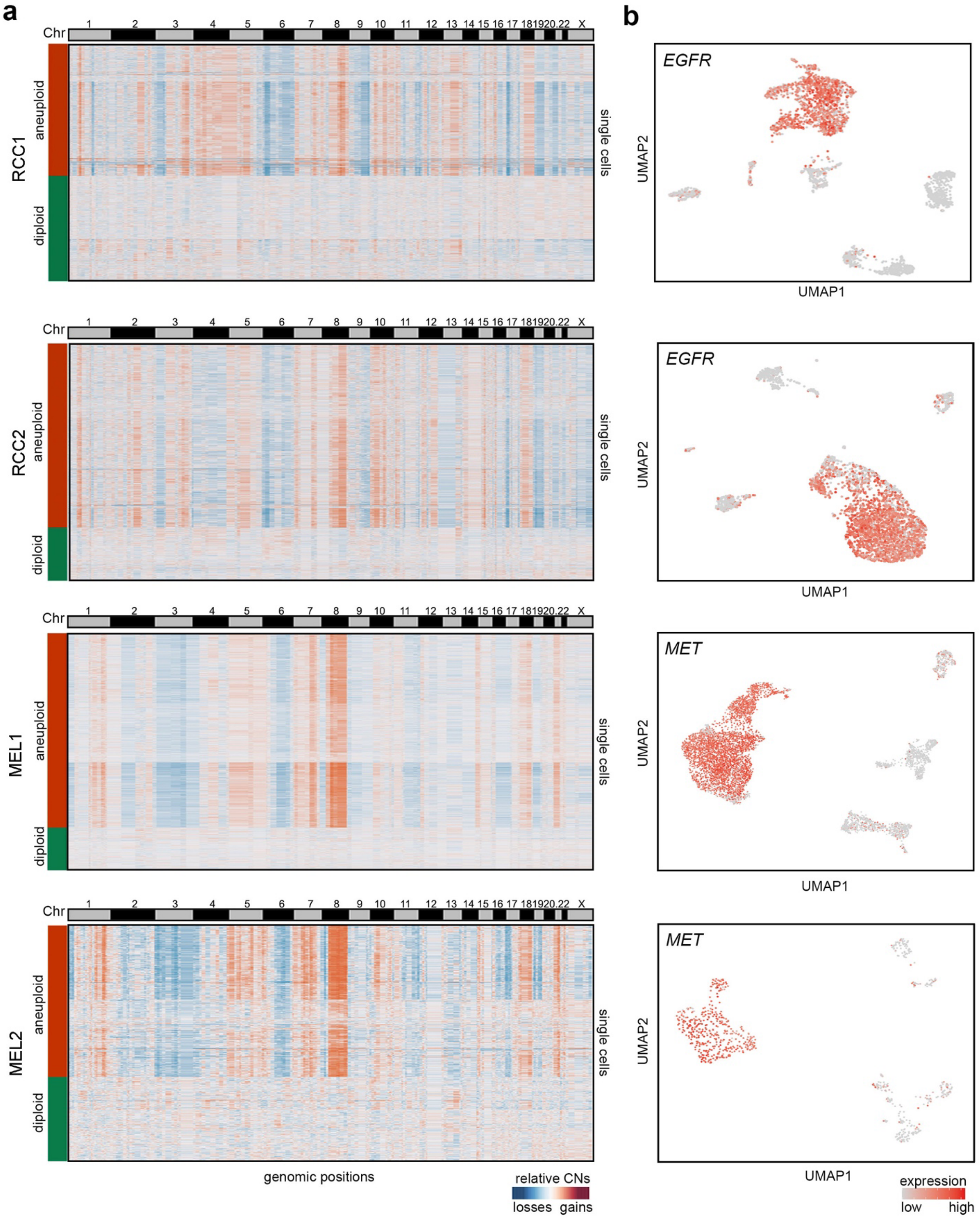

Figure S5 – Classification of tumor and normal cells in tumor samples, related to Figure 3
a, Heatmaps of single cell copy number profiles inferred from scNanoRNAseq data of four frozen tumors. **b**, UMAPs of gene expression levels of known cancer markers, e.g., EGFR in RCCs and MET in melanomas.

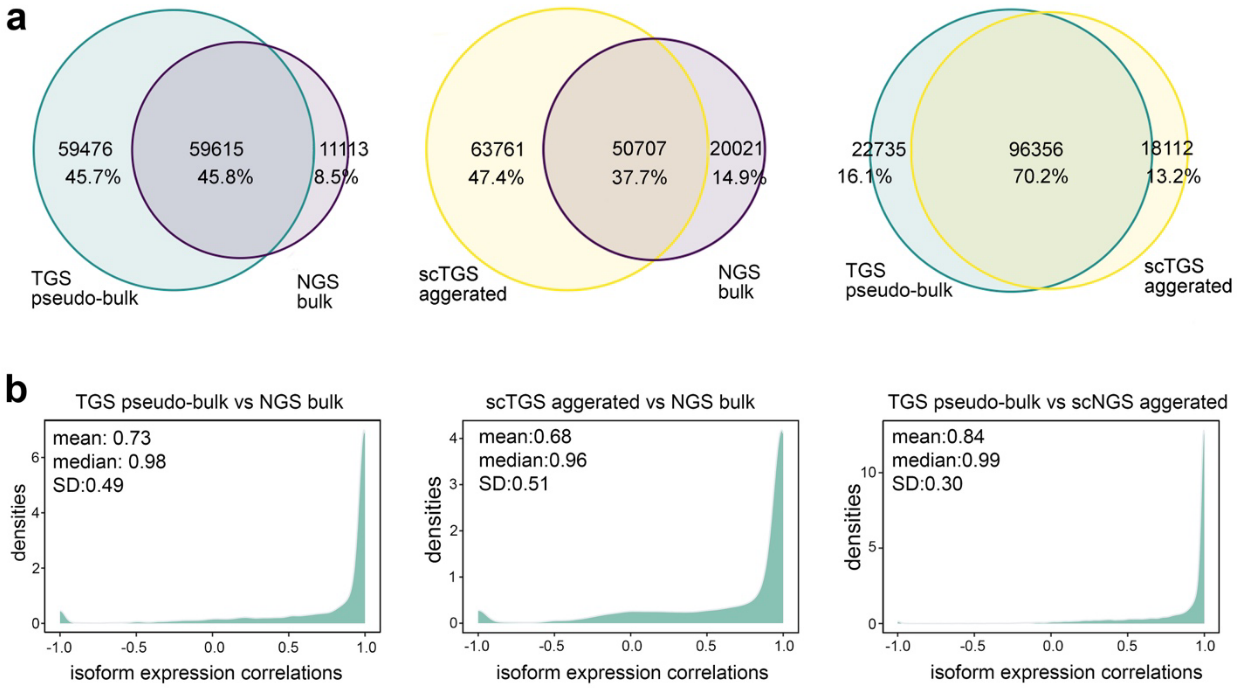

Figure S6 – Comparison of isoforms detected in long and short-read sequencing data of a frozen kidney tumor RCC1, related to Figure 4

a, Ven diagrams of isoforms detected in master FASTQ files of scNanoRNAseq data (TGS pseudo-bulk), traditional NGS-based bulk RNAseq (NGS bulk) and aggregated list of scNanoGPS results (scTGS aggregated). **b**, Density plots of Pearson's correlations of isoform expression levels calculated from the above-mentioned three data types.

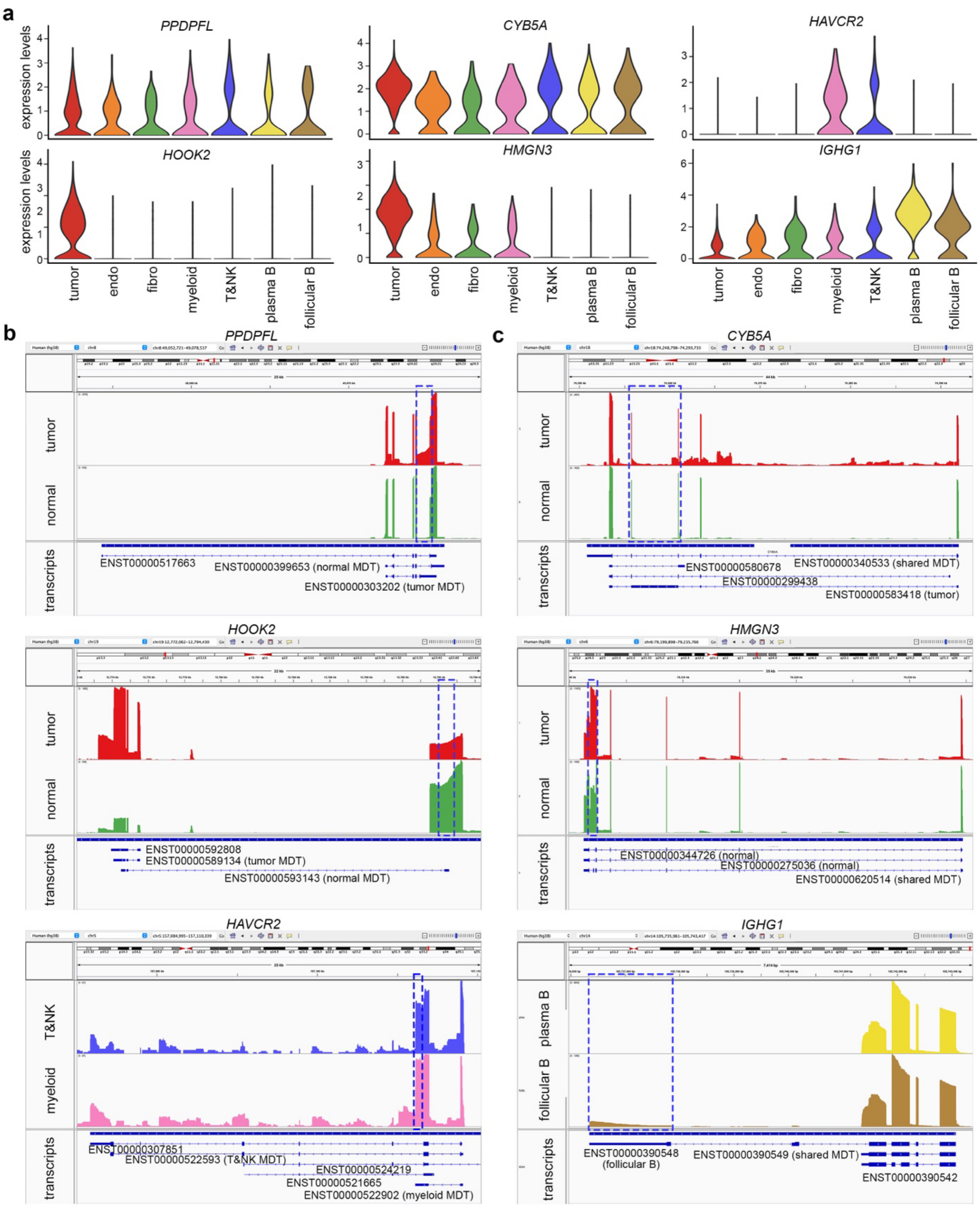

Figure S7 – Cell type specific genes with DCIs, related to Figure 4
a, Violin plots of gene expression levels of 6 example genes with cell-type-specific DCIs. **b**, IGV visualization of reads mapped to 3 example DCIs genes expressing different MDTs in different cell types. **c**, IGV visualization of reads mapped to 3 example DCIs genes expressing same MDTs in different cell types. Shown reads included both pre-mRNA and mRNA mapping reads.

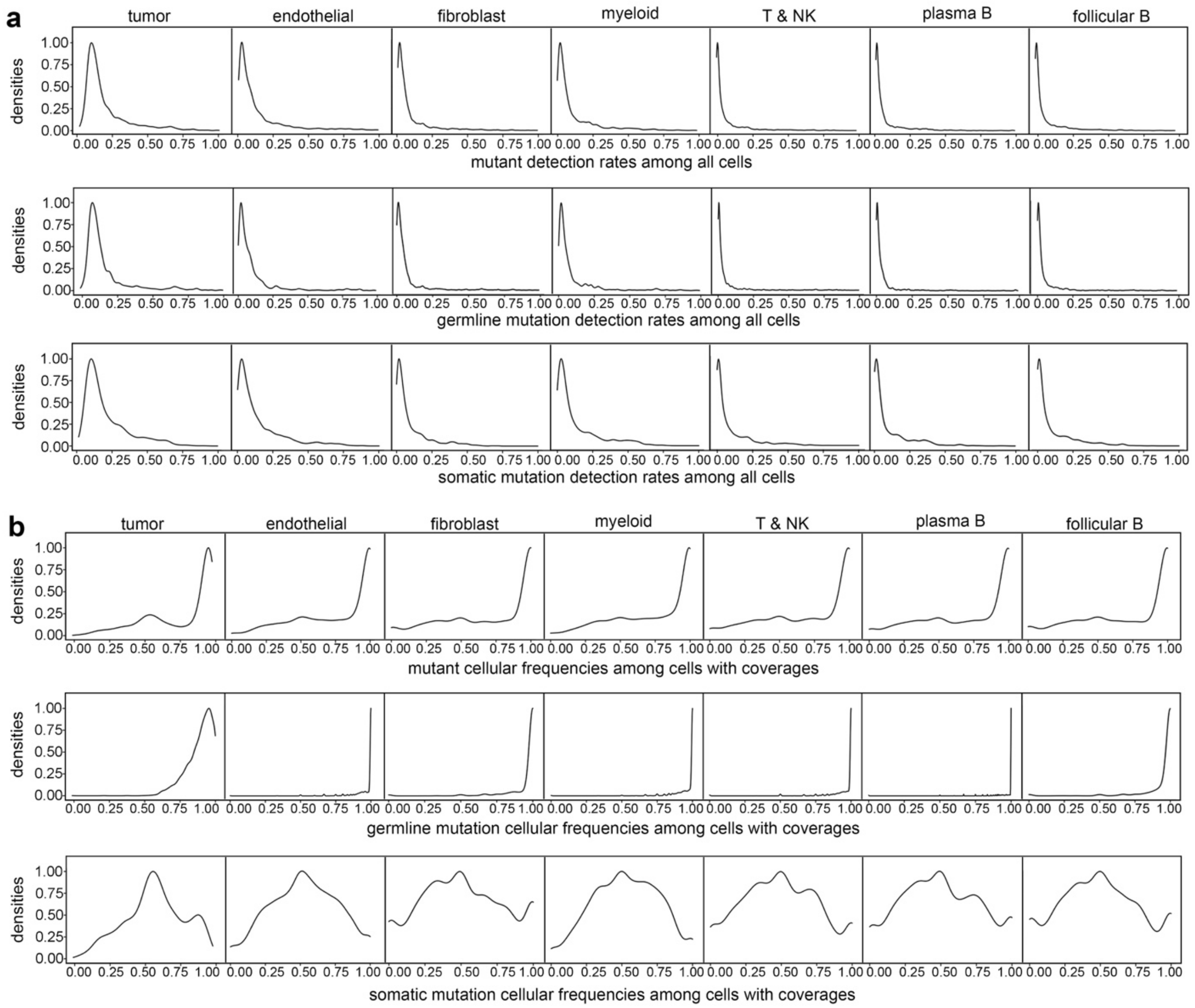

Figure S8 – Mutation detection efficiency and frequencies in different cell types in a frozen kidney tumor RCC1, related to Figure 5

a,Density plots of percentages of cells expressed mutated transcripts overall all cells. **b,** Density plots of cellular frequencies of mutated transcripts over cells with coverages. Top, all point mutations; middle, germline mutations; bottom, somatic mutations.

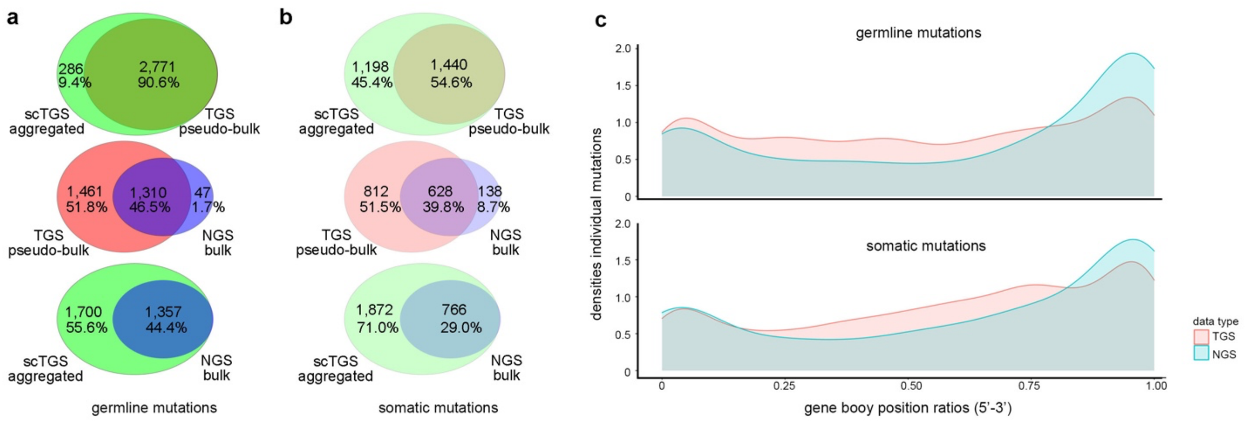

Figure S9 – Comparison of point mutations detected in long and short-read sequencing data of a frozen kidney tumor RCC1, related to Figure 5

Ven diagrams of germline (**a**) or somatic (**b**) mutations detected in master FASTQ files of scNanoRNAseq data (TGS pseudo-bulk), traditional NGS-based bulk RNAseq (NGS bulk) and aggregated list of scNanoGPS results (scTGS aggregated). **c**, Density plots of distributions of detected mutations across relative gene body positions.

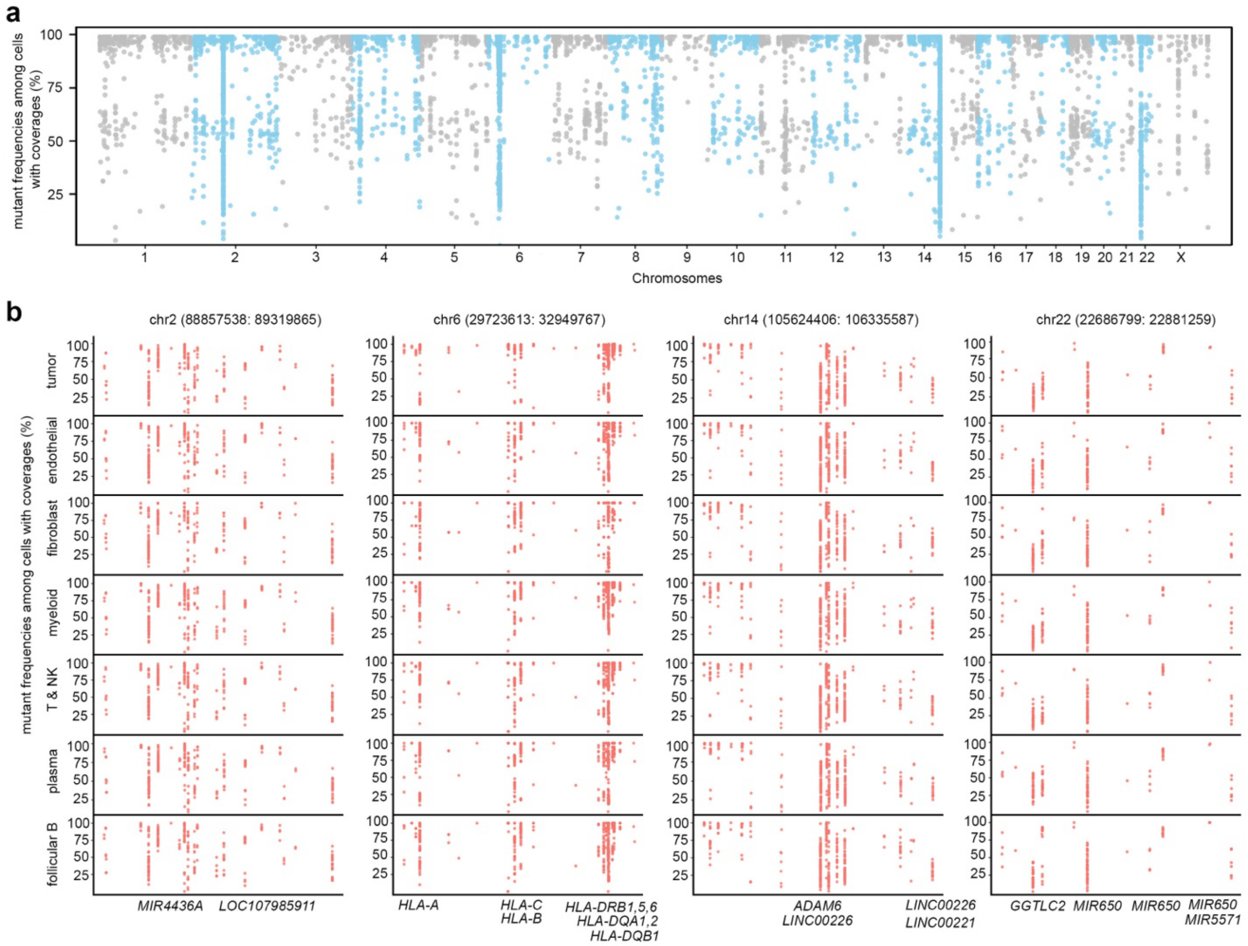

Figure S10 – Shared mutation hotspots in all major cell types from a frozen kidney tumor, related to Figure 5
a, Cellular frequencies of all mutations, i.e., percentages of cells expressed mutants over all cells that had coverages.
b, Cellular frequencies of mutations located in 4 hot spots in each major cell type.

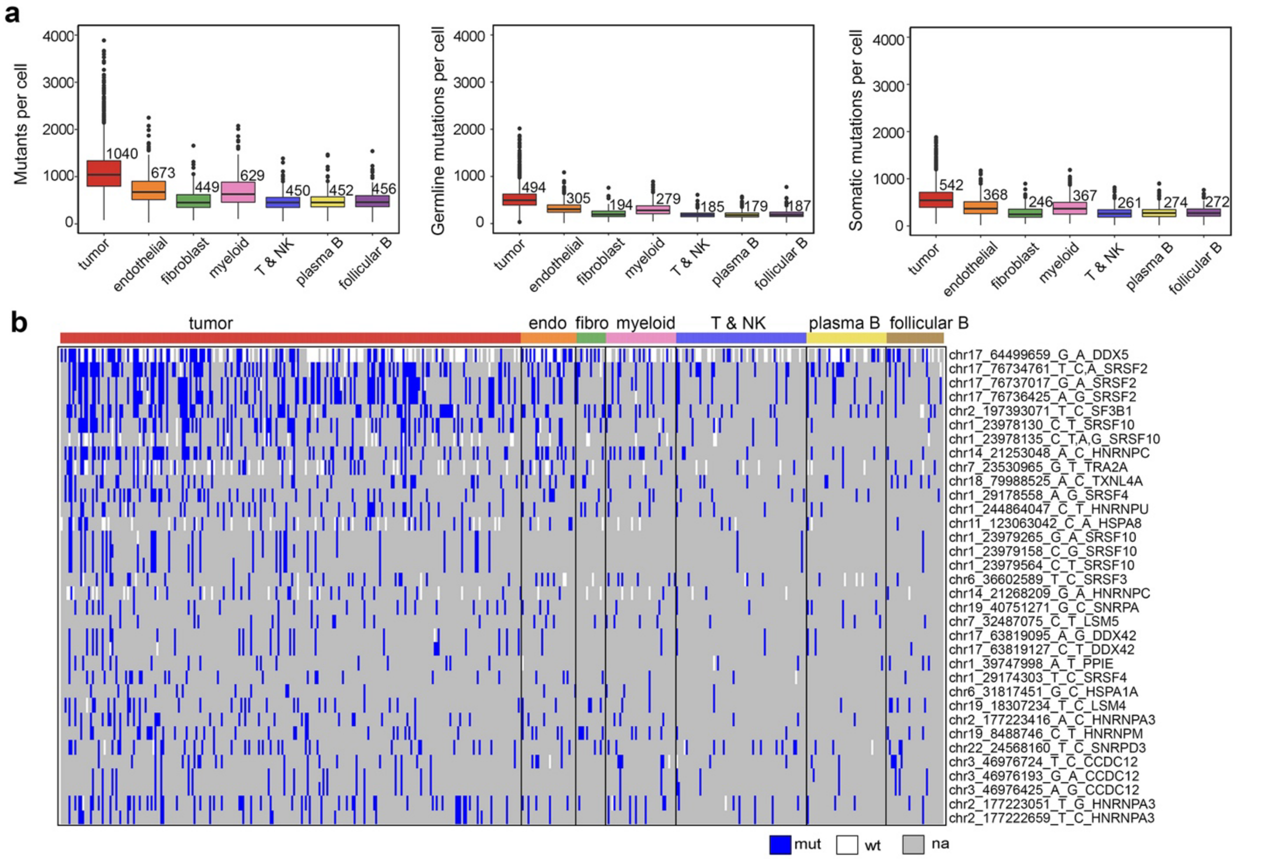

Figure S11 – Single cell transcriptome-wide mutation profiles in a frozen kidney tumor RCC1, related to Figure 5
a, Boxplot showed overall mutations (left), germline mutations (middle), and somatic mutations (right) detected in single cells of each cell type in RCC1. Boxes are centered at median and bounded by first (Q1) and third quartile (Q3). Upper whiskers: minimum of (maximum, Q3 + 1.5 IQR); lower whiskers: maximum of (minimum, Q1 – 1.5 IQR). The median mutation numbers per cells are indicated on top of boxes. **b,** Heatmap of single cell mutations in SPLICESOME genes.

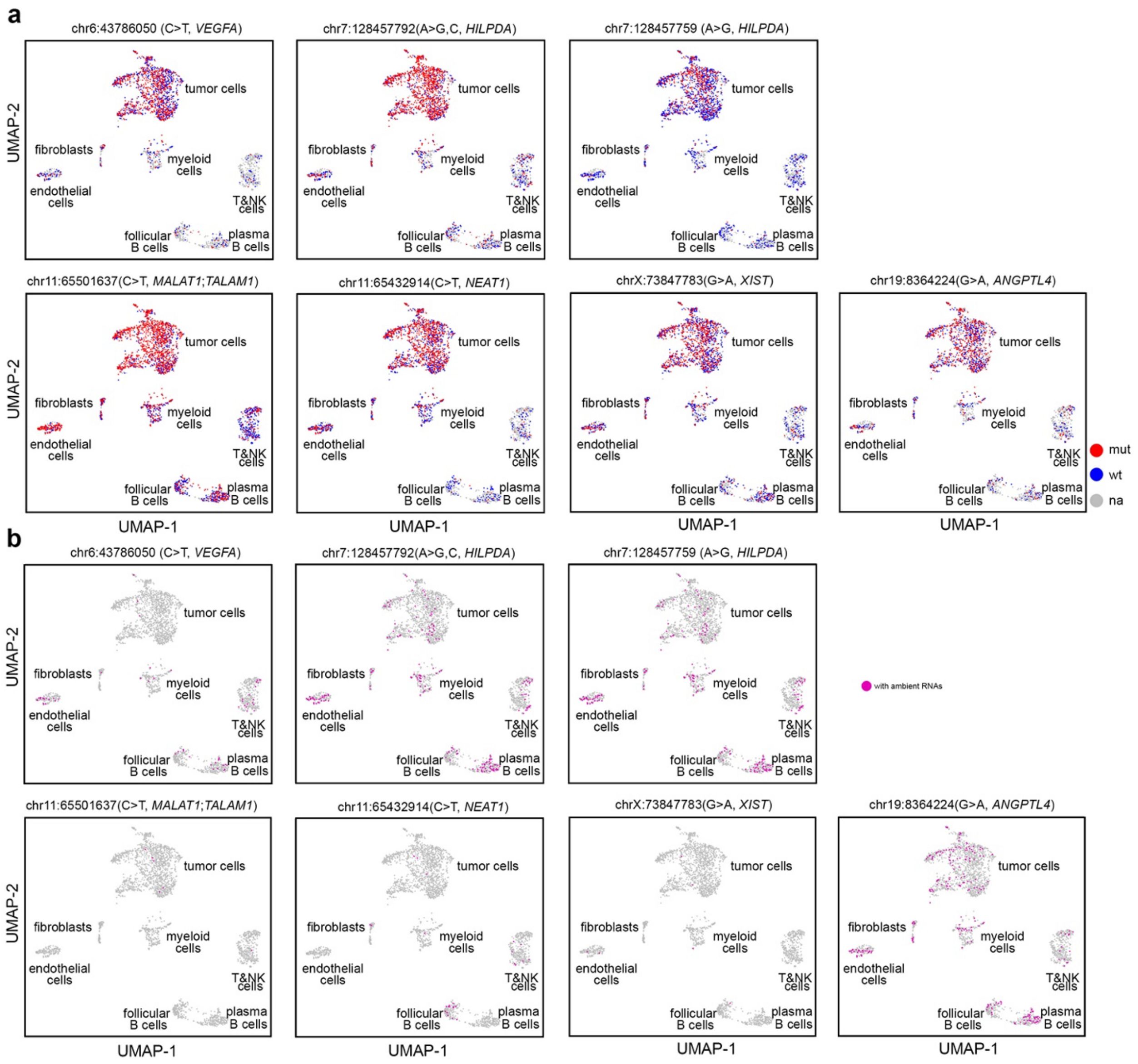

Figure S12 – Examples of tumor-cell-specific deMuts in single cells of a frozen kidney tumor RCC1, related to Figure 5

a, UMAP projections of single cells labeled with 8 examples of tumor-cell-specific deMuts before SoupX. **b**, UMAP projections of single cells with false positive mutation calling affected by ambient RNAs highlighted in magenta.

Supplementary Table 1 - Comparison of functional modules of scNanoGPS with existing tools

Function modules	scNanoGPS	Sockeye	BLAZE
Detection of true CBs	Scan possible CBs from raw data using 4-step iCARLO algorithm; Curate errenous CBs with top-ranked CBs (having more supporting reads); allowing 2 Levenshtein Distance	Compare to barcode whitelist to identify possible CBs, allowing 2 Levenshtein Distance	Compare to barcode whitelist to identify possible CBs, allowing 5 Levenshtein Distance
Detection of UMIs	Mapped to same genomic positions (<5bp), allowing 2 Levenshtein Distance	Network-based clustering, allowing 2 Levenshtein Distance	N/A, by the time of submission
Construction of consensus reads	Collapse reads with same UMIs	N/A, by the time of submission	N/A, by the time of submission
Expression profile calculation	Output gene by cell matrix	Output gene by cell matrix	Output gene by cell matrix
Isoform profile calculation	Output isoform by cell matrix	N/A, by the time of submission	Output isoform by cell matrix
SNV profile calculation	Output SNV by cell matrix	N/A, by the time of submission	N/A, by the time of submission

Supplemental Table 2 - Sample quality metrics

Sample ID	Cancer type	Patient ID	Total reads	Reads past adaptor pattern scanning	Pass rates of adaptor pattern scanning	Median read length	Maximal read length	Averaged quality score in first 100bp	Detected number of cells	Averaged number of raw reads per cell	Exonic mapping rates	Intronic mapping rates	Intergenic mapping rates
RCC1	renal cell carcinoma	RCC12	98,349,656	76,630,302	77.92%	921 bp	190,885 bp	20.47	3,470	22,084	13.91%	70.20%	15.89%
RCC2	renal cell carcinoma - brain metastasis	RCC12	87,800,959	68,137,378	78.83%	830 bp	386,890 bp	20.91	3,638	18,729	15.50%	69.95%	14.55%
MEL1	melanoma	MEL3	72,828,041	55,845,250	78.04%	890 bp	183,138 bp	21.05	7,426	7,520	12.65%	65.92%	21.43%
MEL2	melanoma - brain metastasis	MEL3	98,363,542	75,990,450	78.47%	801 bp	637,942 bp	20.93	1,192	63,750	13.67%	59.62%	26.71%
H2030	non-small cell carcinoma	NA	105,347,205	91,444,891	87.31%	856 bp	411,989 bp	21.81	4,212	21,711	11.84%	59.84%	28.32%
A375	melanoma	NA	67,436,356	58,178,979	86.80%	899 bp	282,743 bp	21.75	3,649	15,944	11.91%	54.91%	33.18%

Supplementary Table 3 - Comparison of CB detection results of three tools

Items	A375			H2030		
	scNanoGPS	BLAZE	Sockeye	scNanoGPS	BLAZE	Sockeye
True positive rate*	97.99%	95.84%	95.85%	98.32%	97.27%	97.23%
False positive rate#	2.01%	4.16%	4.15%	1.68%	2.73%	2.78%
True negative rate	99.91%	99.94%	99.94%	99.93%	99.97%	99.97%
False negative rate	0.90%	0.60%	0.60%	0.70%	0.30%	0.30%
F1 score	0.90	0.92	0.92	0.93	0.96	0.96
Time (H:M:S)	18:17:17	5:01:37	15:37:25	28:02:05	9:40:45	23:10:01
Peak memory	1.21 GB	7.14G	NA	1.30GB	8.31GB	NA

*detected CBs existed in CellRanger list/total detected CBs

#detected CBs not existed in CellRanger list/total detected CBs

Supplementary Table 4 - Usages of computing resources of analyzing A375 data with scNanoGPS

Function Modules*	Time spent (H:M:S)	Memory usage	Storage usage
Input FastQ files	-	-	89 Gb
Scanner	18 : 17 : 17.84	1.30 Gb	52.87 Gb
Assigner	18 : 29 : 35.18	576 Gb	86.50 Mb
Curator	53 : 15 : 38.13	16.82 Gb	74.69 Gb^
Reporter_expression	01 : 49 : 48.48	6.64 Gb	11.65 Mb
Reporter_isoform	11 : 01 : 22.24	2.76 Gb	23.81 Mb
Reporter_SNV	04 : 38 : 56.50	42.55 Gb	12.27 Mb

*, with 30 cores; ^, temporary files

Supplementary Table 5 - Concordance of combinations of CBs, UMIs and genes of scNanoGPS results with CellRanger

Cells	Total combinations of UMIs and Genes (counts)	Overlapped combinations of UMIs and Genes (counts)	Concordance (%)
CCTGCTCCATTAGGCC	6307	4423	70%
GAGCTTAGTTCGCTCA	7541	5288	70%
TTGATGTCATTGTCCT	7995	5567	70%
AGCGGATAGCAGCTAT	11327	7718	68%
GAGCGGTCAGGCTGTT	14193	9889	70%
GATAACGAGCTGTAAC	14696	10103	69%
GCCATGATCACCAATA	19799	13542	68%
CGCTTAACAGGTCCTG	37762	26986	71%
GCAACAGCATGTCAGC	58376	41477	71%
GTATGTGGTATTGAGT	101695	75438	74%
Combinations of CBs, UMIs and Genes (counts)	279691	200431	72%

Supplemental Table 6 - Consensus filtering of RCC1 variants

SNV counts past filtering		Minimal cellular prevalence across all cells						
		0% (1 cell)	0.1% (3 cells)	0.2% (6 cells)	0.4% (13 cells)	0.8% (27 cells)	1% (34 cells)	5% (173 cells)
Minimal consensus reads supporting alternative alleles	1	3,001,137	249,149	86,212	26,895	9,146	6,632	1,004
	2	2,801,998	240,527	83,188	25,939	8,805	6,390*	980
	3	556,867	43,562	15,399	5,960	2,951	2,399	568
	4	121,169	11,979	5,548	2,855	1,678	1,382	383
	5	35,991	6,464	3,424	1,948	1,124	954	296
	6	17,311	4,639	2,634	1,472	870	739	245

*
default

Supplementary Note 1 - Codes of statistical tests

```
##Pearson’s correlations for single cell UMI counts calculated by two approaches in Figure2c
##H2030 is a Seurat object, "RNA" assays store the gene expression results from standard 10X genomics, "NANO" assays store the gene expression results from scNanoGPS
person_cor <- cor.test(H2030$nCount_RNA, H2030$nCount_NANO, method="pearson")$estimate
p_value <-cor.test(H2030$nCount_RNA, H2030$nCount_NANO, method="pearson")$p.value

##Pearson’s correlations for single cell gene expression levels calculated by two approaches in Figure2e
##H2030 is a Seurat object,"RNA" assays store the gene expression results from standard 10X genomics, "NANO" assays store the gene expression results from scNanoGPS
common.genes <- intersect(rownames(H2030@assays$RNA@data), rownames(H2030@assays$NANO@data))
illu<-rowMeans(SN_H2030@assays$RNA@data[common.genes,])
nano<-rowMeans(SN_H2030@assays$NANO@data[common.genes,])
df <- as.data.frame(cbind(illu,nano))
person_cor <- cor.test(df$illu, df$nano, method="pearson")$estimate
p_value <- cor.test(df$illu, df$nano, method="pearson")$p.value

##Paired-two-side t-test for comparing the number of exons of different isoforms of same genes between tumor and normal cells in Figure4d
##df is a data frame, rownames are genes names.
##'tumor_exon_count' column is the number of exons of tumor-cell-preferred isoforms.
##'normal_exon_count' column is the number of exons of normal-cell-preferred isoforms.
p_value<- t.test(df$tumor_exon_count,df$normal_exon_count,paired = T)

##Chi-seq tests to compare the relative frequencies of isoforms in two comparison group
##For each gene, generate a table (df) with n rows (n observations, i.e.,different isoforms) and two columns (two variables,i.e., Group1 and Group2).
##Each cell in the table represents the expreesion level of the corresponding observation (different isoforms) in the corresponding group (Group1 or Group2).
p_value[i] <- chisq.test(df)$p.value

##P-values were adjusted using BH method to adjust for multiple test errors with a false discovery rate of 5%
FDR = p.adjust(p_value, method = "BH")

##Chi-seq tests to compare the relative frequencies of SNVs in two comparison group
##For each SNV, generate a table (df) with two rows (two observations, i.e.,reference and alteration) and two columns (two variables,i.e., Group1 and Group2).
##Each cell in the table represents the number of cells of the corresponding observation (reference or alteration) in the corresponding group (Group1 or Group2).
p_value[i] <- chisq.test(df)$p.value

##P-values were adjusted using BH method to adjust for multiple test errors with a false discovery rate of 5%
FDR = p.adjust(p_value, method = "BH")
```
