# Supplementary material for: High throughput single cell long-read sequencing analyses of same-cell genotypes and phenotypes in human tumors
Source: Nat Commun. 2023 Jul 11;14:4124. doi: 10.1038/s41467-023-39813-7 (PMC10336110; doi:10.1038/s41467-023-39813-7)
Supplement: Supplementary file 2 — Description of Additional Supplementary Files [file 41467_2023_39813_MOESM2_ESM.pdf]

Description of Additional Supplementary Files

Supplementary Data 1 - Tumor cell specific DCI genes Description: This table includes tumor-cell-specific DCI genes that express different combinations of isoforms in tumor cells comparing to normal cell types in the same tumor.

Supplementary Data 2 - Endothelial cell specific DCI genes Description: This table includes endothelial-cell-specific DCI genes that express different combinations of isoforms in endothelial cells comparing to other normal cell types in the same tumor.

Supplementary Data 3 - Myeloid cell specific DCI genes Description: This table includes myeloid-cell-specific DCI genes that express different combinations of isoforms in myeloid cells comparing to other normal cell types in the same tumor.

Supplementary Data 4 – Follicular B cell specific DCI genes Description: This table includes follicular-cell-specific DCI genes that express different combinations of isoforms in follicular B cells comparing to other normal cell types in the same tumor.

Supplementary Data 5 – Lymphocyte specific DCI genes Description: This table includes lymphocyte-cell-specific DCI genes that express different combinations of isoforms in T/B cells comparing to other normal cell types in the same tumor.

Supplementary Data 6 – Plasma cell specific DCI genes Description: This table includes plasma-cell-specific DCI genes that express different combinations of isoforms in plasma cells comparing to other normal cell types in the same tumor.

Supplementary Data 7 – Fibroblast specific DCI genes Description: This table includes fibroblast-cell-specific DCI genes that express different combinations of isoforms in fibroblasts comparing to other normal cell types in the same tumor.

Supplementary Data 8 – Tumor cell expanded mutations Description: This table includes point mutations that are significantly expanded in tumor cells comparing to normal cell types in the same tumor.

Supplementary Data 9 – Myeloid cell expanded mutations Description: This table includes point mutations that are significantly expanded in myeloid cells comparing to other normal cell types in the same tumor.

Supplementary Data 10 – Plasma cell expanded mutations Description: This table includes point mutations that are significantly expanded in plasma cells comparing to other normal cell types in the same tumor.

Supplementary Data 11 – Endothelial cell expanded mutations Description: This table includes point mutations that are significantly expanded in endothelial cells comparing to other normal cell types in the same tumor.

Supplementary Data 12 – Follicular B cell expanded mutations Description: This table includes point mutations that are significantly expanded in follicular B cells comparing to other normal cell types in the same tumor.

Supplementary Data 13 – Lymphocytes expanded mutations Description: This table includes point mutations that are significantly expanded in T/B cells comparing to other normal cell types in the same tumor.

Supplementary Data 14 – Fibroblast expanded mutations Description: This table includes point mutations that are significantly expanded in fibroblasts comparing to other normal cell types in the same tumor.
