# Supplementary material for: High throughput single cell long-read sequencing analyses of same-cell genotypes and phenotypes in human tumors
Source: Nat Commun. 2023 Jul 11;14:4124. doi: 10.1038/s41467-023-39813-7 (PMC10336110; doi:10.1038/s41467-023-39813-7)
Supplement: Supplementary file 4 — Reporting Summary [file 41467_2023_39813_MOESM4_ESM.pdf]

nature portfolio

Corresponding author(s): Ruli Gao

Last updated by author(s): Jun 3, 2023

Reporting Summary

Nature Portfolio wishes to improve the reproducibility of the work that we publish. This form provides structure for consistency and transparency in reporting. For further information on Nature Portfolio policies, see our Editorial Policies and the Editorial Policy Checklist.

Statistics

For all statistical analyses, confirm that the following items are present in the figure legend, table legend, main text, or Methods section.

n/a	Confirmed	
☐	☒	The exact sample size (*n*) for each experimental group/condition, given as a discrete number and unit of measurement
☐	☒	A statement on whether measurements were taken from distinct samples or whether the same sample was measured repeatedly
☐	☒	The statistical test(s) used AND whether they are one- or two-sided<br>*Only common tests should be described solely by name; describe more complex techniques in the Methods section.*
☐	☒	A description of all covariates tested
☐	☒	A description of any assumptions or corrections, such as tests of normality and adjustment for multiple comparisons
☐	☒	A full description of the statistical parameters including central tendency (e.g. means) or other basic estimates (e.g. regression coefficient) AND variation (e.g. standard deviation) or associated estimates of uncertainty (e.g. confidence intervals)
☐	☒	For null hypothesis testing, the test statistic (e.g. *F*, *t*, *r*) with confidence intervals, effect sizes, degrees of freedom and *P* value noted<br>*Give P values as exact values whenever suitable.*
☐	☒	For Bayesian analysis, information on the choice of priors and Markov chain Monte Carlo settings
☐	☒	For hierarchical and complex designs, identification of the appropriate level for tests and full reporting of outcomes
☐	☒	Estimates of effect sizes (e.g. Cohen's *d*, Pearson's *r*), indicating how they were calculated

Our web collection on statistics for biologists contains articles on many of the points above.

Software and code

Policy information about availability of computer code

Data collection	No software or code was used to collect data.
Data analysis	We used base-caller guppy (v5.0.12), CellRanger ARC (v2.0), FastQC (v0.12.0), MiniMap2(v2.26), SPOA(v4.0.7), Samtools (v1.15), dbSNP (v150), COSMIC (v96), CopyKAT(v1.0.6), ANNOVAR, DoubletFinder(v2.0.3), our own software, scNanoGPS (https://github.com/gaolabtools/scNanoGPS). Reference databases used in this study include GENCODE (v32) and human reference genome GRCh38.

For manuscripts utilizing custom algorithms or software that are central to the research but not yet described in published literature, software must be made available to editors and reviewers. We strongly encourage code deposition in a community repository (e.g. GitHub). See the Nature Portfolio guidelines for submitting code & software for further information.

Data

Policy information about availability of data

All manuscripts must include a data availability statement. This statement should provide the following information, where applicable:

- Accession codes, unique identifiers, or web links for publicly available datasets
- A description of any restrictions on data availability
- For clinical datasets or third party data, please ensure that the statement adheres to our policy

All raw single cell sequencing data and processed gene expression matrix have been submitted to Gene Expression Omnibus (GEO): GSE212945 and are made publicly available. There is no restriction on data.

Research involving human participants, their data, or biological material

Policy information about studies with human participants or human data. See also policy information about sex, gender (identity/presentation), and sexual orientation and race, ethnicity and racism.

Reporting on sex and gender	This study doesn't report any data or results related to sex and gender.
Reporting on race, ethnicity, or other socially relevant groupings	This study doesn't report any data or results related to any socially relevant groupings.
Population characteristics	This study doesn't report any data or results related to population characteristics.
Recruitment	This study doesn't involve recruitement.
Ethics oversight	Northwestern University Biomedical IRB board.

Note that full information on the approval of the study protocol must also be provided in the manuscript.

Field-specific reporting

Please select the one below that is the best fit for your research. If you are not sure, read the appropriate sections before making your selection.

☒ Life sciences ☐ Behavioural & social sciences ☐ Ecological, evolutionary & environmental sciences

For a reference copy of the document with all sections, see nature.com/documents/nr-reporting-summary-flat.pdf

Life sciences study design

All studies must disclose on these points even when the disclosure is negative.

Sample size	Our own single cell Nanopore RNA sequencing data that was generated based on the availability of surgical samples and cell lines. This study does not depend on specific statistical tests to establish significance for patient numbers. Therefore power calculations are not required to establish sample size.
Data exclusions	A subset of single cells with low quality were excluded from the study during a QC filtering step, which excluded cells with insufficient sequencing reads or gene detection . The specific filtering criteria used during these steps is stated in the methods sections.
Replication	The single cell nanopore RNA sequencing experiments were performed on 4 frozen human tissue samples and 2cultured cell lines, which were considered as biological replications. All samples are included in this study.
Randomization	There are no experimental groups in this study.
Blinding	There are no experimental groups in this study.

Reporting for specific materials, systems and methods

We require information from authors about some types of materials, experimental systems and methods used in many studies. Here, indicate whether each material, system or method listed is relevant to your study. If you are not sure if a list item applies to your research, read the appropriate section before selecting a response.

Materials & experimental systems

n/a	Involved in the study	
☒	☐	Antibodies
☐	☒	Eukaryotic cell lines
☒	☐	Palaeontology and archaeology
☒	☐	Animals and other organisms
☒	☐	Clinical data
☒	☐	Dual use research of concern
☒	☐	Plants

Methods

n/a	Involved in the study	
☒	☐	ChIP-seq
☒	☐	Flow cytometry
☒	☐	MRI-based neuroimaging

Eukaryotic cell lines

Policy information about cell lines and Sex and Gender in Research

Cell line source(s)	A375 is provided by Dr. Michael A. Davies at MD Anderson Cancer Center. H2030 is provided by the Antibody and Bioresource Core Facility at Memorial Sloan Kettering. Material Transfer Agreements are approved.
Authentication	Authentication of both cell lines are performed in the Cytogenetics and Cell Authentication Core at MD Anderson Cancer Center.
Mycoplasma contamination	Both cell lines are tested negative of mycoplasma contamination.
Commonly misidentified lines (See ICLAC register)	Neither cell line is listed.
